# Physical mechanisms governing generalization and hallucination in deep learning for imaging through scattering media

**DOI:** 10.1038/s41467-026-72304-z

**Published:** 2026-04-23

**Authors:** Xuyu Zhang, Tianting Zhong, Haofan Huang, Dawei Zhang, Songlin Zhuang, Shensheng Han, Puxiang Lai, Honglin Liu

**Affiliations:** 1 Wangzhijiang Innovation Center for Laser, Aerospace Laser Technology and System Department, Shanghai Institute of Optics and Fine Mechanics, Chinese Academy of Sciences, Shanghai, China; 2 School of Optical-Electrical and Computer Engineering, University of Shanghai for Science and Technology, Shanghai, China; 3 Department of Biomedical Engineering, The Hong Kong Polytechnic University, Hong Kong SAR, China; 4 Center of Materials Science and Optoelectronics Engineering, University of Chinese Academy of Science, Beijing, China; 5 Photonics Research Institute, The Hong Kong Polytechnic University, Hong Kong SAR, China; 6 The Hong Kong Polytechnic University Shenzhen Research Institute, Shenzhen, China

**Keywords:** Imaging and sensing, Computational science

## Abstract

Deep learning has revolutionized computational imaging, yet its real-world deployment remains constrained by two critical challenges: poor generalization under dynamic conditions and the emergence of hallucinatory artifacts. By leveraging a physics-guided framework based on scattering media, a model system where controlled variations in light transmission matrices ($T$) isolates these challenges, we unravel the mechanistic interplay between generalization limits and hallucination origins. We demonstrate that a network’s generalization capacity is fundamentally bounded by its ability to accommodate distinct inverse mappings (${T}^{-1}$), while hallucinations arise when this capacity is exceeded, resulting in unconstrained, non-physical predictions. We also identify residual ballistic light, if not negligible, as a stabilizing anchor, enabling robust predictions under scattering variability. Integrating experimental validation with wave-optics simulations, we establish a universal framework that links these phenomena, showing that strategic training on diverse physical mappings enhances generalization while suppressing hallucinations. This work bridges physics-driven interpretability with AI design, offering actionable strategies to develop reliable models for applications ranging from medical imaging through biological tissues to autonomous navigation in scattering environments.

## Abstract (web summary)

Deep learning for computational imaging faces the challenges of poor generalization under dynamic conditions and hallucinatory artifacts. The authors use a physics-guided scattering model to reveal hallucinations emerge when networks cannot handle diverse optical inverse mappings. They show that training on varied physical mappings improves generalization and suppresses hallucinations.

## Introduction

Deep learning has emerged as a transformative tool of artificial intelligence (AI), achieving breakthroughs in tasks from computer vision, natural language processing, medical diagnosis, financial technology, environmental science, and autonomous navigation^1–8^. However, its transition from controlled demonstration to real-world applications remains hindered by two persistent challenges: generalization, the ability to perform robustly under unseen/untrained conditions^9–11^, and hallucination, the generation of erroneous, ungrounded predictions^12–15^.

These issues are particularly acute in computational imaging, where dynamic physical environments (e.g., scattering media, fluctuating illumination) demand models that adapt to ever-shifting system mappings. Current solutions, such as adversarial training or dataset augmentation^16–18^, often treat these challenges as purely algorithmic, neglecting the physical principles governing the data generation process. Here, we bridge this gap by establishing a physics-driven framework to decode the origins of generalization and hallucination, using imaging through scattering media as a paradigmatic model.

At its core, an imaging system can be mathematically formulated as $Y={TX}$, where $X$ denotes the input target, $Y$ corresponds to the observed output (e.g., a speckle pattern), and $T$ encapsulates the intrinsic physical response of the system. In scattering media, $T$ is highly sensitive to perturbations such as refractive index variations or mechanical displacements, causing drastic changes in the input-output relationship^19–23^. The key to resolving the cross-target generalization challenge across disparate datasets lies in learning a mapping that closely approximates the system’s intrinsic physical transformation, enabling universal imaging of diverse targets irrespective of their specific features. To achieve this objective, the training dataset must be optimized to ensure that every pixel on both the input and output planes is uniformly and sufficiently sampled, in strict accordance with the principle of spacetime homogeneity^24^. Remaining generalization challenges can be largely attributed to $T$. Specifically, a neural network’s generalization capability is determined by its capacity to identify multiple distinct system matrices $\{{T}_{i}\}$ —or equivalently, the number of unique inverse mappings $\{{T}_{i}^{-1}\}$ it can effectively accommodate. While generalization in deep learning conventionally implies adaptability across both the data domain ($X$) and the system mapping domain ($T$), existing research has focused predominantly on enhancing generalization performance. In contrast, this work aims to unveil the fundamental physical mechanisms governing this behavior. Since $T$ and $X$ represent distinct physical factors, this study focuses primarily on generalization across the $T$ domain.

After training network under specific conditions, conventional deep learning approaches can learn an approximate mapping relationship $M$, which can approach ${T}^{-1}$ infinitely in principle^24^, to predict the image^25–27^. But their performance collapses when $T$ deviates beyond a narrow range^25,26^. This limitation stems from a critical oversight: neural networks are typically designed to learn a single inverse mapping ${T}^{-1}$, while real-world systems exhibit a continuum of $T$ matrices. One intuitive solution to improve the network’s generalization is to train the network to simultaneously recognize multiple *Ts*. Correspondingly, the generalization range can be expanded through consecutive arrangements of multiple mappings by proper selections. We hypothesize that a network’s generalization capacity is dictated by its ability to accommodate multiple ${T}^{-1}$ mappings, and hallucinations arise when the diversity of *T* exceeds this capacity, forcing the network into unconstrained, non-physical predictions. It should be noted that in this study, we are not referring to the generalization from unseen scattering media based on residual ballistic light, whose $T$ is constant^28^.

**Fig. 1:**
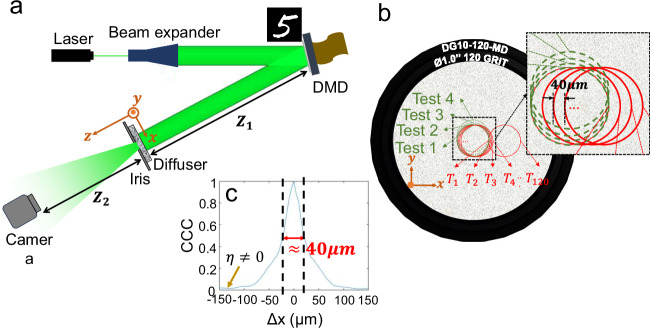
Experimental setup and transmission matrix (*T*) characterization. **a** Schematic of the lensless imaging system: a laser illuminates a DMD displaying MNIST digits; scattered light from the diffuser forms speckle patterns recorded by a CCD camera. **b** Training data regions (${T}_{1}$–${T}_{120}$, red solid circles) generated by 40 μm lateral shifts of the diffuser. Testing regions (Tests 1–4, green dash circles) are vertically shifted from ${T}_{1}$ by 10, 20, and 50 μm, respectively. ${T}_{120}$ partially overlaps ${T}_{1}$ due to the relatively large circular illumination area (shown in dashed zoom). **c** Cross-correlation coefficient (CCC) of speckle patterns versus lateral displacement, revealing a 40 µm isoplanatic range (red arrow) beyond which *T*′ becomes uncorrelated from *T*.

To test the hypothesis above, we leverage a tunable optical system (Fig. 1a), where $T$ is controllably varied by laterally shifting a scattering diffuser—a lensless imaging setup that isolates $T$-dependent effects from confounding variables such as field-of-view or resolution changes. There is no coupling between different physical variables; the transmission matrix $T$ can be altered simply by changing the refractive index distribution within the illuminated region, but other factors remain constant. By training a convolutional neural network (CNN) on datasets spanning distinct $T$ regimes, we quantify how generalization scales with the network’s $T$-accommodation capacity and identify the threshold beyond which hallucinations dominate. Crucially, we disentangle the roles of scattered and ballistic light components, revealing how residual ballistic photons stabilize predictions under $T$-variability—a phenomenon previously attributed solely to scattered light statistics^29–32^.

In this work, we establish a universal relationship between a network’s physical “bandwidth” (number of $T$ matrices it can accommodate) and its generalization-hallucination trade-off. Our findings demonstrate that hybrid training on multi-$T$ datasets, combined with ballistic-light priors, enhances robustness without sacrificing accuracy. Further, by correlating network behavior with wave-optics physics, we move beyond “black-box” explanations, offering a physics-grounded roadmap for trustworthy AI in dynamic environments. This work not only addresses critical barriers in computational imaging but also provides a template for physics-driven AI design, with implications for autonomous navigation in fog, underwater imaging, and medical diagnostics through biological tissues.

## Results

### Training strategy optimization

**Fig. 2:**
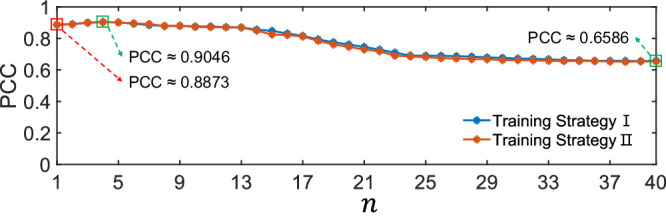
Experimental validation of two training strategies. Performance comparison of two training strategies with experimental data, where the prediction accuracy (Pearson Correlation Coefficient, PCC) is plotted versus the number of accommodated transmission matrices (*n*).

We first compared two training strategies to balance computational efficiency and prediction accuracy. As shown in Fig. 2, Strategy I (batch training on $n$ × 5000 samples) and Strategy II (sequential training on incremental $T$ matrices) achieved comparable prediction accuracy. That said, Strategy II significantly reduced training time, demonstrating that iterative refinement of $T$-specific mappings does not compromise performance. Therefore, Strategy II was adopted for subsequent network training. Note that beyond $n$ = 20, both strategies exhibited declining PCC due to the network’s finite capacity to accommodate distinct ${T}^{-1}$ mappings, confirming our first hypothesis. Due to the presence of residual ballistic light, the PCC finally stabilized around 0.65, enabling successful image prediction even when $n$ = 40 and proving that ballistic residue is the key to the generalization capability^28^.

### Ballistic light stabilizes predictions under *T*-variability

**Fig. 3:**
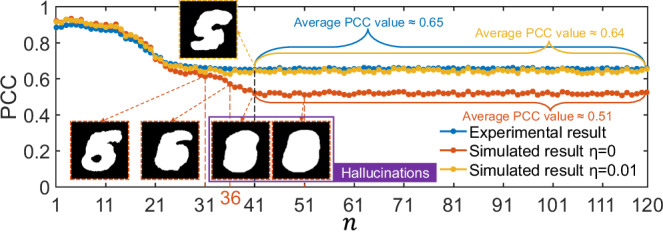
Ballistic light stabilizes network predictions under varying transmission matrices. Experimental and simulated PCC versus the number of accommodated $T$ matrices ($n$). For $\eta$ = 0.01, simulations (yellow curve) match experiments (blue curve), with PCC stabilizing at ~0.65 due to ballistic light. For $\eta$ = 0 (red curve), PCC collapses at $n$ > 30, and the images reconstructed at $n$ = 31, 36, 41, and 51 fail to recover the target objects. Here, Test 1 data is exclusively used across varying $n$ values to evaluate the network’s capacity to accommodate different *Ts*. The decay of PCC is purely caused by the increase of *n*, rather than the diminished overlap between the test and reference areas.

To isolate the roles of scattered and ballistic light, we trained networks on simulated data with controlled ballistic proportions ($\eta$). Based on the cross-correlation coefficient curve in Fig. 1c, whose tail is higher than 0, the proportion of ballistic residue after passing through the diffuser is estimated to be $\eta \approx 0.01$. As shown in Fig. 3, when $\eta$ = 0.01 (matching experimental residual, blue curve), predictions as represented by the yellow curve and boxed reconstruction remained robust even at $n$ = 40 (PCC > 0.6). But when there is no ballistic light at all, i.e., $\eta$ = 0, complete failure beyond $n$ = 30 is seen, as represented by the red curve and the four boxed reconstructions corresponding to $n$=31, 36, 41, and 51. This divergence highlights a critical insight: ballistic light provides a stable inverse mapping (${T}_{{ballistic}}^{-1}$) that anchors predictions when scattered light mappings (${T}_{{scattered}}^{-1}$) become incompatible.

To validate the universality of our findings, we replicated the experiments using a generative adversarial network (GAN). A qualitatively consistent phenomenon was observed, with the only notable variation being the specific threshold value of $n$. These results further confirm that the generalization limitations of deep learning models stem from inherent physical constraints rather than the specific algorithmic deficiencies (see Section A in Supplementary Information).

Furthermore, while data augmentation generally improves the fidelity with which simulated mappings approximate true physical mappings, we found that it exerts a negligible effect on the network’s capacity to accommodate diverse independent mappings. The detailed experimental results supporting this conclusion are presented in Section B of the Supplementary Information.

### Generalization boundaries and Hallucination threshold

**Fig. 4:**
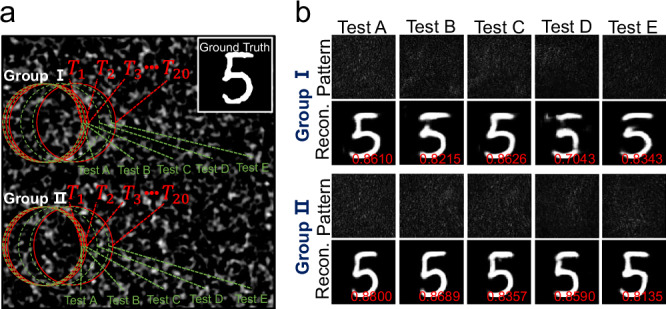
Generalization boundaries of the network trained with multiple transmission matrices. **a** Illumination regions for training (red solid circles, ${T}_{1}$ – ${T}_{20}$) and testing (green dash circles, Tests A–E) with lateral shifts of 40 µm (Group I) or 30 µm (Group II). **b** Reconstructed images (Digit “5”) for Tests A–E, showing retained fidelity (PCC > 0.7).

To quantify the network’s generalization capacity to scattering matrices, it is necessary to eliminate the influence of ballistic components. Figure 4 shows the predicted imaging results of the network in a paired comparison when it is trained to accommodate 20 different *Ts* in the absence of ballistic residue with a modified tactic in data acquisition. In Group I, ${T}_{i+1}$ is laterally shifted 40 μm to the right of ${T}_{i}$, and five different regions were tested. Test A coincides with the region of ${T}_{1}$, while Tests B, C, D and E are shifted to the right of ${T}_{1}$ by 0.5×40 μm, 2.3×40 μm, 9.6×40 μm and 18.2×40 μm, respectively, with the latter four positions randomly selected within the scanning range from ${T}_{1}$ to ${T}_{20}$. The network successfully predicted images (PCC > 0.7) through all five positions, demonstrating generalization across the range of ${T}_{1}$ to ${T}_{20}$. In Group II, ${T}_{i+1}$ was shifted 30 μm to the right of ${T}_{i}$, and five different regions were tested, too. Test A coincides with the region of ${T}_{1}$, while Tests B, C, D, and E were shifted 0.5 × 30 μm, 2.3 × 30 μm, 9.6 × 30 μm, and 18.2 × 30 μm to the right of ${T}_{1}$, respectively. Similar to Group I, the network could successfully image (PCC > 0.8) at any position within the scan range. However, due to partial correlation between ${T}_{i}$ and ${T}_{i+1}$, the actually accommodated independent mapping relationships was less than 20, which results in generally better imaging quality compared to Group I. Obviously, by carefully selecting mapping relationships, the network’s generalization can be further improved, ensuring reliable predictions over a broader range, i.e., enhancing the generalization of the network.

**Fig. 5:**
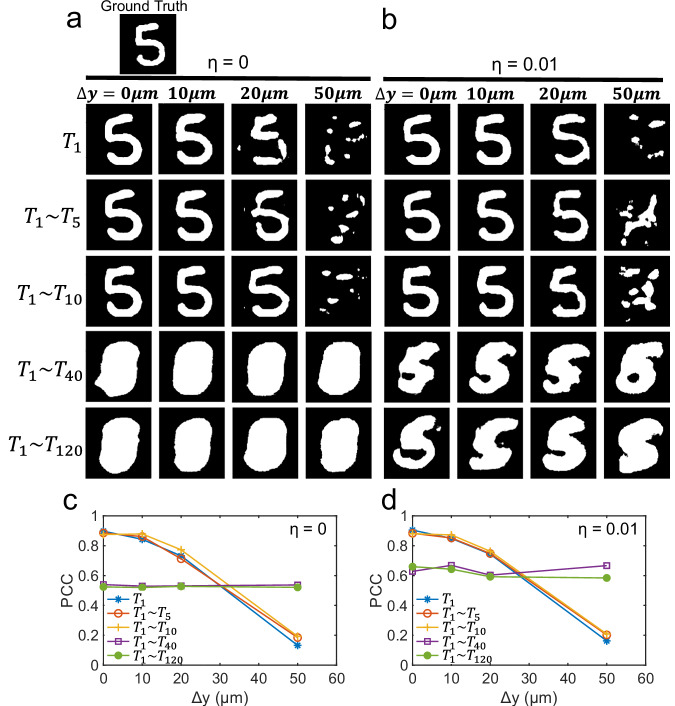
Hallucinations emerge when training exceeds network’s compatibility. **a** Reconstructed images for increasing numbers of transmission matrices ($n$) without ballistic light *(*$\eta$ = 0). Beyond $n$ = 20, artifacts dominate, culminating in hallucinations (e.g., $n$ = 40). Within the generalization range, the reconstructed image quality deteriorates as $\Delta y$ increases. When $\Delta y$= 50 µm, reconstruction fails. **b** With $\eta$ = 0.01, ballistic light stabilizes predictions, especially with a large number of transmission matrices being trained, enabling partial feature retention even at $\Delta y$ = 50 µm. **c**, **d** PCC versus vertical shift ($\Delta y$) for varying number of transmission matrices when $\eta$ = 0 (c) and $\eta$ = 0.01 (**d**).

In the above results, all testing matrices are seen or partially seen during training. In Fig. 5 we compared the results of network predictions from seen to unseen matrices under varying numbers of independent $T$ by shifting the testing area vertically, *i.e*., changing $\Delta y$ as shown in Fig. 1b. As seen, without ballistic residue (Fig. 5a), the network can still reconstruct images within the permissible number of independent $T$ ($n$ ≤ 20), though the image qualities in Test 1 ($\Delta y=0\,\mu m$) declined slightly with increased $n$. In other words, targets can be reconstructed under good constraints. The network’s capability rapidly deteriorated from Test 1 to 4. At $\Delta y=20\,\mu m$, near the edge of the original generalization range, the reconstruction was merely successful; at $\Delta y=50\,\mu m$, the reconstruction failed. This also presents direct evidence that the transmission matrix $T$ of Test 4 is independent from ${T}_{1}$–${T}_{120}$, despite the presence of various overlaps. Note that at $\Delta y=50\,\mu m$, there were effective relationships learned in the network, and there was target information in the speckles, but the network could not decode it due to the mismatch between encoding and decoding, i.e., predictions fail due to impropriate constraints. If there was no target information in speckles, the same phenomena appeared, too.

When the number of independent $T$ exceeded the network’s compatibility ($n$ > 20), hallucinations emerged as incoherent patterns, as shown in the fifth and sixth rows of Fig. 5a. These hallucinations arise due to the network’s failure to learn any effective mapping relationship, i.e., too many compromises were made for all $T$ matrices, resulting in no constraints in prediction. Strikingly, these artifacts preserved structural priors (e.g., centered digits), revealing that hallucinations arise from unconstrained mappings rather than random noise. When a small amount of ballistic component was introduced ($\eta=0.01$ in Fig. 5b), the reconstructions became recognizable, since the network had learned the mapping relationship of the ballistic component, allowing for information extraction from the ballistic residue. Meanwhile, the hallucination from the scattering component (specifically due to the severely compromised relationship learned from scattered light) persisted, creating artifacts in the reconstructed images. For results of more digits, please refer to Section C in Supplementary Information.

Increasing the proportion of ballistic component can reduce and eventually eliminate these artifacts. As an effective mapping relationship emerges, the hallucinations diminish or even disappear. In this situation, the network can simultaneously learn mappings for both scattering and ballistic components, enabling accurate information reconstruction. With fewer *Ts*, the network primarily extracts information from the scattering component, but as the number of *T* increases, the network’s ability to extract images from the ballistic component is strengthened. Since the ballistic component is almost unaffected by displacement, the network’s sensitivity to position variation decreases, yielding enhanced generalization to see through unlearned scattering media^28^. Detailed theoretical analyses of network prediction under diverse physical conditions are provided in Section D of the Supplementary Information.

### Phase transition between generalization and hallucination

**Fig. 6:**
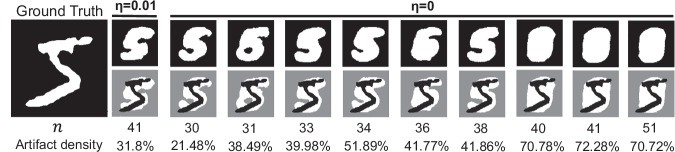
Hallucination progression with increasing transmission matrices. Reconstructed images (top) and overlays (bottom) for $\eta$ = 0 show escalating artifacts as *n* exceeds the network’s compatibility limit *(*$n$ > 20). Artifact density (white) increases from 21% (*n* = 30) to >70% ($n$ = 40), rendering predictions unrecognizable. With $\eta$ = 0.01, ballistic light suppresses artifacts by >45%, retaining discernible features even at $n$ = 41.

Increasing $n$ beyond the network’s compatibility limit ($n$ > 20) triggered a sharp transition: predictions shifted from physically grounded reconstructions to hallucinations dominated by artifacts (Fig. 6). At $n$ = 40, artifact density exceeded 70% for $\eta$ = 0, rendering images unrecognizable. However, introducing $\eta$ = 0.01 suppressed artifacts by ~45%, enabling partial recovery of target features (Fig. 6, lower row). This phase transition underscores a fundamental trade-off: expanding $T$-accommodation enhances generalization but risks hallucinations unless stabilized by physical priors (e.g., ballistic light).

The artifact density is defined as the percentage of bright pixels in the binarized reconstruction image whose values deviate from the ground truth. Mathematically, it is expressed as:

$${D}_{{artifact}}=\frac{\mathop{\sum }\nolimits_{\left(i,j\right)\in {{\rm{N}}}_{{gt}}}I\left(\left|{Y}_{{pred}}\left(i ,j\right)-{Y}_{{gt}}\left(i,j\right)\right|\ge 0.5\right)}{\mathop{\sum }\nolimits_{\left(i,j\right)\in {{\rm{N}}}_{{gt}}}I\left(\left|{Y}_{{gt}}\left(i,j\right)\right|\ge 0.5\right)}\times 100\%,$$

where ${Y}_{{pred}}$ and ${Y}_{{gt}}$ represent the binarized reconstruction image and ground-truth image, respectively; ${N}_{{gt}}$ is the total pixel count in$\,{Y}_{{gt}}$; $I\left(\cdot \right)$ is an indicator function, which takes a value of 1 when the internal condition holds, and 0 otherwise. For a sanity check of the results of the preset threshold, please refer to Section E in Supplementary Information.

### Universal scaling of generalization capacity

By testing overlapping and non-overlapping $T$ shifts (Fig. 4), we established that the network’s compatibility limit scales with the independence of $T$ matrices, not their total number. For partially correlated $T$ matrices (*e.g*., 30 µm shifts), the network accommodated 30% more mappings while maintaining PCC > 0.7, demonstrating that strategic *T*-selection can optimize generalization without architectural changes.

## Discussion

This study reveals a fundamental trade-off governing deep learning in dynamic physical systems: generalization scales with a network’s capacity to accommodate diverse physical mappings, while hallucinations emerge when this capacity is exceeded. We distinguish here between two distinct classes of error. The first, which is the focus of this work, arises when the network is forced to accommodate valid physical mappings beyond its structural capacity; in this regime, enhanced generalization paradoxically correlates with increasingly severe hallucinations due to compromised internal relationships. The second class stems from source-reference divergence, artifacts caused by deficient mappings learned from imperfect or noisy data. As noted in Ref.^24^, errors of this second type can be suppressed by improving generalization (i.e., learning a relationship that asymptotically approaches the true mapping). However, regardless of the origin, hallucinations ultimately reflect a limited or imperfect understanding of underlying reality. Collectively, these mechanistic insights resolve long-standing debates regarding whether hallucinations stem fundamentally from model limitations^33,34^ or data imperfections^35,36^.

We elaborate here on the core mechanism where hallucinations occur when system diversity exceeds network capacity. Notably, this mechanism is applicable to a wide range of dynamic scattering scenarios, including dense fog and seawater. Its practical implications are threefold: First, it guides network architecture design, for example, informing the decision to develop hybrid networks trained on both ballistic and scattered light signals. Second, it provides a rigorous framework for evaluating training data collection strategies, specifically determining the requisite sample density across varying physical states to sustain generalization. Third, it enables the quantitative assessment of upper performance bounds for distinct application scenarios. A critical advance of this study is the quantification of generalization as a function of the independence of physical mappings (i.e., transmission matrices $T$), rather than merely their quantity. This insight redefines dataset design principles: instead of amassing arbitrary variations, the strategic selection of maximally distinct *T* matrices (determined, for example, by isoplanatic range thresholds) optimizes generalization. Ultimately, such physics-guided dataset curation holds transformative potential for resource-intensive real-world applications, such as autonomous navigation in fog, where high-cost field data collection poses a major bottleneck.

For different diffusers, their isoplanatic ranges and proportions of residual ballistic light $\eta$ may differ. However, the fundamental principles obtained through our study are general. For instance, a volumetric scattering medium can be considered as ballistic-free when its optical thickness is sufficiently large, whereas phase plates or coarse-grit diffusers^26^ may minimize but not eliminate ballistic residue. The ballistic component’s stabilizing role (e.g., $\eta$ = 0.01) highlights a broader principle: physical priors act as anchors to suppress hallucinations without sacrificing generalization. This mirrors biological systems like the human visual cortex, which integrates redundant sensory cues (e.g., motion parallax, stereopsis) to stabilize perception in cluttered environments^37,38^. Our framework extends this principle to AI, proposing that hybrid architectures combining physics-based invariants (e.g., ballistic light, diffraction symmetries) with data-driven adaptability can overcome the scalability limits of current models.

In this study, we focused on the network’s ability to accommodate mappings for imaging through scattering media. In other imaging applications, such as point-to-point geometric imaging or Fresnel diffraction imaging, the corresponding mappings exhibit significantly lower complexity. While this inherently imposes less stringent requirements on the network’s learning-to-simulate capability, the underlying physical principles remain consistent. Consequently, the evaluation methodology proposed here can be extended to assess generalization limits in these lower-complexity applications as well.

Furthermore, while we validated our framework in optics, its principles, such as compatibility thresholds and anchor priors, are likely universal. In natural language processing, for example, domain-specific invariants (e.g., grammar rules) may serve as an analogous role in mitigating hallucinations when models generalize across dialects^39–41^. Neural networks must comply with a variety of constraints, beyond linguistic ones, to generate outputs that are both structurally correct and consistent with real-world facts. If these underlying principles are not adequately modeled, distortions and hallucinations will inevitably emerge.

Finally, despite the immeasurable complexities and infinite source requirements, it remains impossible to integrate every principle of nature and rule of humanity into an AI model, as many remain unknown. Consequently, for an “open” model operating in the real world, hallucinations can be mitigated but are unlikely to be eliminated. This stands in distinct contrast to “closed” spaces, such as the board games Chess and Go, where all rules are accurately defined and bounded. By treating neural networks not as black boxes but as physical systems governed by compatibility thresholds, this work paves the way for interpretable, physics-grounded AI.

In summary, this work establishes a physics-driven framework to decode the origins of generalization and hallucination in deep learning, using imaging through scattering media as a paradigmatic model. We demonstrate that generalization is fundamentally bounded by a network’s capacity to resolve distinct inverse mappings (${T}^{-1}$), whereas ballistic light priors extend this limit by stabilizing anchoring for prediction. Critically, we reveal that hallucinations emerge when the diversity of physical mappings exceeds the network’s capacity for accommodation, leading to the generation of unconstrained yet structured artifacts. Finally, we confirm that physics-aware training based on strategic $T$-selection and hybrid architectures can enhance network robustness without compromising accuracy. Aiming to bridge the gap between AI and physical sciences, this work offers a blueprint for designing trustworthy models in dynamic environments. Treating neural networks not as opaque “black boxes” but as *physical systems* governed by definable compatibility thresholds paves the way for the development of interpretable, reliable AI in applications ranging from medical imaging through biological tissues to autonomous navigation in scattering environments. Future efforts will extend this framework to nonlinear and multi-physics systems, leveraging domain-specific invariants to navigate the delicate balance between adaptability and fidelity.

## Methods

### Experimental setup

The optical system (Fig. 1) consisted of a solid-state laser (MGL-III-532-200mW, $\lambda=532{nm},$ Changchun New Industries Optoelectronics Tech) whose beam was expanded to illuminate a digital micromirror device (DMD, V-7001 VIS, 1024 × 768 pixels, 13.7 µm/pixel, ViALUX). Light reflected from the DMD passes through a ground glass diffuser (DG10−120-MD, 120-grit, Thorlabs) and forms a speckle pattern recorded by a CCD camera (Ace acA2440–75um, 2448 × 2048 pixels, 3.45 μm/pixel, Basler). The object-image distance was set at $u=16{cm}$ and $v=10{cm}$, respectively. Handwritten digits from the MNIST database^42,43^ were resized to 64 × 64 arrays and displayed sequentially at the center of the DMD. An iris (5 mm diameter) controlled the illumination zone on the diffuser, resulting in an average speckle size of 10.64 μm on the camera plane.

### Mathematical formulation of speckle pattern retrieval in deep learning

In our experimental setup, the recorded speckle pattern is described as $Y={\rm{| }}{TX}{{\rm{| }}}^{2}$, a nonlinear relationship. The retrieval process can thus be modeled as $X={T}^{-1}{\mathscr{R}}Y$, where ${\mathscr{R}}$ represents the intensity-to-field transformation, and ${T}^{-1}$ denotes the inverse of the linear transmission matrix $T$. A neural network is capable of simultaneously learning both the linear inverse mapping ${T}^{-1}$ and the nonlinear transformation ${\mathscr{R}}$. Notably, ${\mathscr{R}}$ remains constant across distinct ${T}^{-1}$ s. To simplify the subsequent theoretical analysis, the nonlinear component is neglected.

### Uncorrelated speckle patterns and transmission matrix independence

An intensity distribution measured under single fixed-angle coherent illumination does not, by itself, constitute a full transmission matrix. Nevertheless, if two forward mappings (i.e., recorded speckle patterns) corresponding to different diffuser shifts are mutually uncorrelated, their respective transmission matrices can be considered independent.

**Fig. 7:**
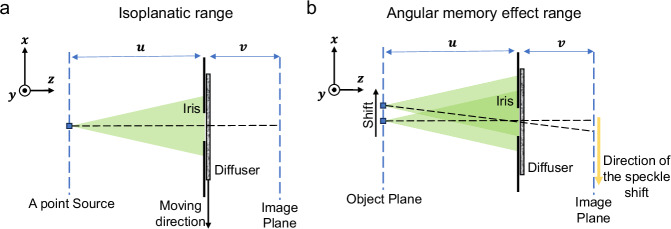
Schematic illustration for establishing transmission matrix (*T*) independence. **a** Measuring the isoplanatic range (translational memory effect): a lateral diffuser shift beyond this range yields uncorrelated speckle patterns (and thus independent complex fields). **b** Constructing the transmission matrix: each column corresponds to the complex field from a point source within the field of view. Uncorrelated fields from different diffuser shifts imply independent *T* matrices.

As illustrated in Fig. 7a, when a point source illuminates a diffuser, a speckle pattern—or equivalently, a complex field—is obtained. When the diffuser is laterally shifted, a new speckle pattern (and its corresponding complex field) is generated. Once the shift exceeds the isoplanatic range (equivalent to the translational memory effect range), the two speckle patterns are mutually uncorrelated, implying that their corresponding complex fields share this uncorrelated property.

In Fig. 7b, shifting the point source across the object plane within the angular memory effect range results in a speckle pattern (and complex field) that maintains an almost identical distribution, differing only by a spatial translation. The transmission matrix is constructed by assembling the complex fields from all point-source positions within the field of view (FOV). Given that the complex-field components arising from different diffuser shifts are mutually uncorrelated, we can rigorously conclude that the corresponding transmission matrices are independent. This conclusion holds even when the point source shift exceeds the angular memory effect range. Especially, when the object distance $u$ is sufficiently large, the measurement setup effectively reduces to the case of single fixed-angle coherent illumination, consistent with the configuration shown in Fig. 1a.

### Data acquisition and transmission matrix variation

The standard MNIST dataset (download from https://yann.lecun.com/exdb/mnist/; it is open access and could be used with permission for research and academic dissemination) was partitioned into two subsets: images #1-50,000 for training and images #50,001–60,000 for testing. For each transmission matrix $T$ in the training set, 5000 targets were randomly selected to obtain the corresponding speckle patterns. Testing targets were drawn exclusively from the testing subset to ensure they were never encountered during the training phase. We observed that the network prediction performance tends to stabilize once the number of training sample pairs reaches approximately 3000 per $T$.

To probe the network’s generalization limits, we systematically varied the scattering transmission matrix $T$ by laterally displacing the diffuser. When the illumination region was shifted within 20 μm from the center, the new mapping relationship was close to the original one, that is to say, ${corr}(T,{T}^{{\prime} })\ge 0.5$, and the network retained its generalization ability. The isoplanatic range (40 μm), the displacement threshold beyond which $T$ becomes uncorrelated, was calibrated via cross-correlation analysis of speckle patterns (Fig. 1c). Training datasets included *n* distinct *T* matrices (${T}_{1}$, ${T}_{2}$, …, ${T}_{n}$), each generated by shifting the diffuser in 40 μm increments (red solid circles in Fig. 1b). In experiment, we tried up to ${T}_{120}$, which resulted in a total lateral shift of $\left(120-1\right)\times 40\,\mu m=4.76{mm}$. For each $T$, 5000 speckle-target pairs were collected for training, ensuring sufficient data to learn a mapping relationship. Testing datasets included four additional groups (Tests 1-4), where the illumination region of Test 1 coincided with ${T}_{1}$ and Tests 2-4 were shifted along the y-axis by 10, 20, and 50 μm from Test 1, respectively (green dash circles in Fig. 1b).

### Training strategies

As the number of mapping relationships in the training set increases, the data volume, and consequently, the training time, rise rapidly. Two strategies were employed to train a 5-layer U-Net architecture (Tensorflow2/Keras, NVIDIA RTX3090)^44^:

### Strategy I (batching training)

The network was trained from scratch on $5000\times n$ pairs across all $n$ matrices.

### Strategy II (sequential training)

Each time a new transmission matrix $T$ was added, the next 5000 pairs of data were used to continuously train the network. This is to say, the network corresponding to ${nT}$ was incrementally trained by adding new 5000 pairs onto the network already trained for $(n-1)T$.

Both strategies used a batch size of 10, and the time required for each epoch was about 49 s; when the number of training samples doubled, the training time also doubled. Prediction accuracy was quantified using the Pearson Correlation Coefficient (PCC)^45,46^ between reconstructed and ground-truth images. The time consumption of Strategy II is proportional to $n$; for Strategy I it is proportional to $n(n+1)/2$.

### Wave-optics simulation

Usually, there is a small amount of ballistic light residual when a light beam passes through a diffuser, and the transmission matrix of the ballistic component does not change with refractive index variations in the illuminated region. In an experiment, it is challenging to eliminate ballistic residuals or to control their proportion. Therefore, in addition to experimental data, we also included simulation data for training and testing, allowing the proportion of ballistic light to be precisely controlled. Based on wave optics, we simulated speckle patterns for each digit image. The ground glass diffuser was represented as a random phase screen exp[−*jCϕ*(*x, y*)], where the phase $\phi (x,y)$ followed a Gaussian distribution^28,47^. The quantitative relationship between $C$ and $\eta$ was calibrated beforehand, and the ballistic component’s proportion $\eta$ was controlled by tuning $C$, enabling direct comparison with experimental data. We selected the specific value of $C$ for which the tail of the normalized cross-correlation curve obtained via simulation matches that derived from experimental measurements. Speckle intensity was calculated as light field emitted from the target plane, transmitted through the random phase screen, and finally reaching the detection plane. The free-space propagation between adjacent planes was described by the Rayleigh-Sommerfeld equation.

### Network compatibility testing

The network’s capacity to accommodate distinct $T$ matrices was evaluated by training on datasets with increasing $n$. Generalization was tested on laterally shifted *T*′ matrices outside the training distribution (Fig. 4a). To isolate scattered light effects, simulations with $\eta=0$ (no ballistic component) were compared to $\eta$=0.01, matching experimental ballistic residuals.

## Supplementary information

Supplementary Information

Transparent Peer Review file

## Data Availability

The raw data generated in this study are publicly accessible via the GitHub repository at https://github.com/XuyuZhang98/Code-for-imaging-through-scattering-media-based-on-deep-learning.git.

## Code availability

The code employed in this study is publicly available in the open-access GitHub repository https://github.com/XuyuZhang98/Code-for-imaging-through-scattering-media-based-on-deep-learning.git.
