# Supplementary material for: Physical mechanisms governing generalization and hallucination in deep learning for imaging through scattering media
Source: Nat Commun. 2026 Apr 23;17:5616. doi: 10.1038/s41467-026-72304-z (PMC13315912; doi:10.1038/s41467-026-72304-z)
Supplement: Supplementary file 1 — Supplementary Information [file 41467_2026_72304_MOESM1_ESM.pdf]

# Supplementary Information for "Physical Mechanisms Governing Generalization and Hallucination in Deep Learning for Imaging through Scattering Media"

Xuyu Zhang(张栩瑜),[1,2,+] Tianting Zhong(仲天庭),[3,+] Haofan Huang(黄浩梵),[3,+] Dawei Zhang(张大伟),[2] Songlin Zhuang(庄松林),[2] Shensheng Han(韩申生),[1,4] Puxiang Lai(赖溥祥),[3,5,6,*] and Honglin Liu(刘红林),[1,4,*]

[1]Wangzhijiang Innovation Center for Laser, Aerospace Laser Technology and System Department, Shanghai Institute of Optics and Fine Mechanics, Chinese Academy of Sciences, Shanghai 201800, China

[2]School of Optical-Electrical and Computer Engineering, University of Shanghai for Science and Technology, Shanghai 200093, China

[3]Department of Biomedical Engineering, The Hong Kong Polytechnic University, Hong Kong SAR, China

[4]Center of Materials Science and Optoelectronics Engineering, University of Chinese Academy of Science, Beijing 100049, China

[5]Photonics Research Institute, The Hong Kong Polytechnic University, Hong Kong SAR, China

[6]The Hong Kong Polytechnic University Shenzhen Research Institute, Shenzhen, China

[+]Equal contribution

*Corresponding authors: puxiang.lai@polyu.edu.hk, and hlliu4@hotmail.com

## Section A. Physical Constraints rather than Algorithmic Deficiencies Underlie Network Generalization Limitations

A prevailing hypothesis in deep learning research suggests that the generalization capability of a model can be enhanced simply by adopting more sophisticated network architectures. However, this work contends that the generalization limitations observed in our experiments stem primarily from inherent physical constraints rather than algorithmic inadequacies. Specifically, when a deep learning model is tasked with handling a number of transmission matrices (TMs) that exceeds the its processing capacity, the model's generalization performance degrades drastically, and hallucination artifacts emerge.

This underlying physical principle is corroborated by results obtained using a generative adversarial network (GAN), as presented in **Fig. S1**. Compared to the 5-layer U-Net used in our primary study, the GAN architecture can accommodate a larger number of independent $T$ matrices. However, once the number of independent matrices exceeds the GAN's specific capacity limit (in this case, $n = 61$), the generated images exhibit hallucinations analogous to those seen in the U-Net. These findings strongly support the core conclusion of our paper: generalization is capacity bounded.

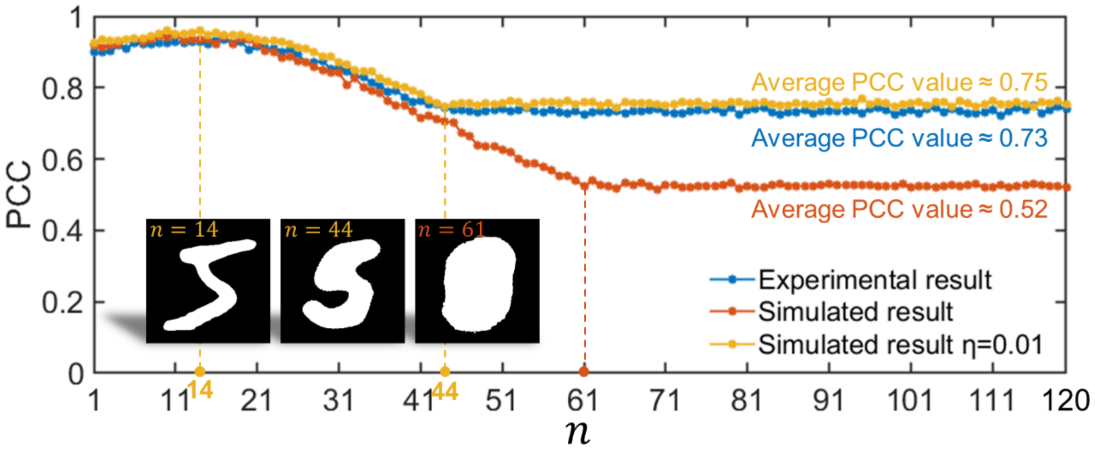


Fig. S1. Accommodation capability test of the GAN with and without ballistic light. The GAN accommodates more independent transmission matrices ($T$) compared to the 5-layer U-Net, but hallucinations similarly emerge when $n$ (here, $n = 61$) exceeds the network's capacity.

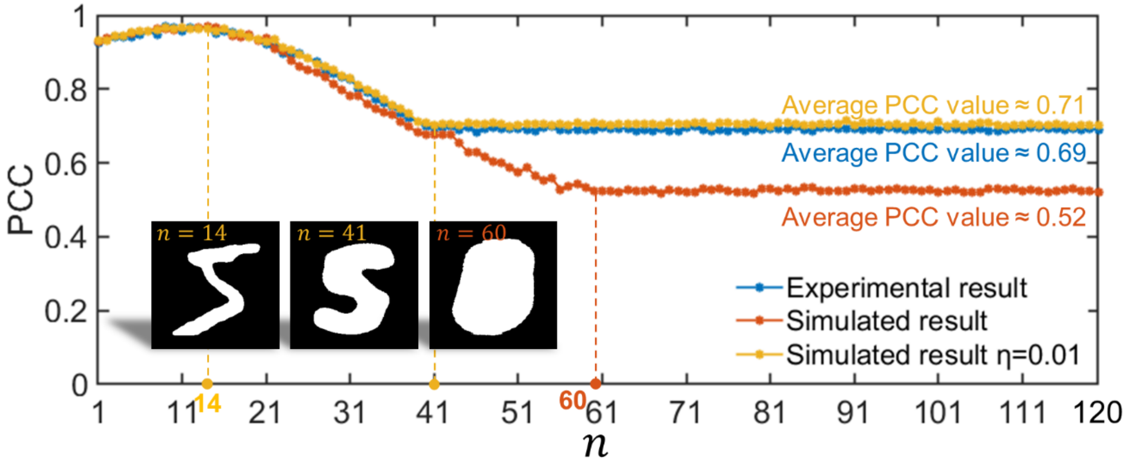


Fig. S2 Accommodation capability test of the 6-layer U-Net with and without ballistic light.

Analogous behavior is observed using a 6-layer U-Net, as shown in Fig. S2. In comparison with its 5-layer counterpart, the 6-layer architecture exhibits a stronger capacity to accommodate independent $T$s ($n > 41$). However, hallucinations still emerge once this extended capacity is surpassed.

Overall, while wider and deeper networks generally improve performance, albeit requiring larger training datasets and longer training cycles, they remain subject to the same fundamental constraints. Under a fixed training paradigm, a network's generalization ability is defined by its finite capacity to accommodate distinct physical mappings. Regardless of the specific architecture or scale, exceeding this capacity threshold triggers hallucinations, highlighting the universality of the physical constraints identified in this study.

**Section B. Comparison of the U-Net Accommodation Capability with and without Data Augmentation**

We further evaluated the efficacy of contrast enhancement as a data augmentation strategy within our scattering-medium imaging framework. Experiments were conducted using the identical 5-layer U-Net architecture. As illustrated in Fig. S3, while contrast enhancement yields a marginal improvement in image quality (reflected in slightly higher PCC values across all $n$ values when compared against the reference data shown in Fig. 4), it produces only a negligible enhancement in the U-Net's capacity to accommodate different $T$s.

These results reinforce our core premise: neural networks inherently possess a finite capacity for accommodating independent mapping relationships. As the number of mappings $n$ increases, image quality degrades progressively, distortions become increasingly severe, and hallucinations eventually emerge. This aligns with the conclusion of Ref. [24] that data augmentation serves primarily to enhance generalization within the domain of $X$ (i.e., simulating a specific mapping more accurately) rather than expanding the capacity to resolve distinct mappings in the domain of $T$.

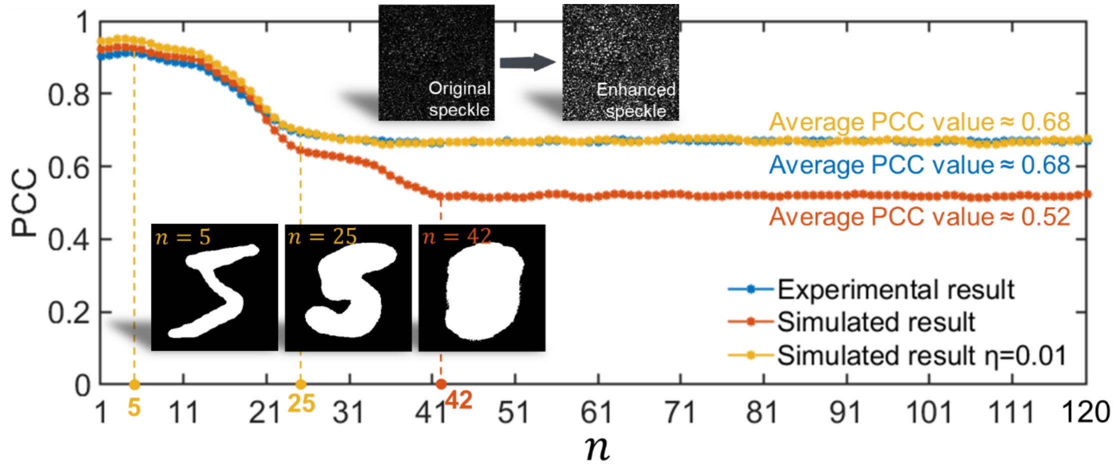


Fig. S3 Results of the 5-layer U-Net's accommodation capability for independent $T$s under contrast enhancement of recorded speckle patterns.

**Section C. Comparison of Imaging Results for Different Digits**

To verify robustness, the trained U-Net was employed to reconstruct images of diverse targets (digits 0-9) under varying experimental conditions, as illustrated in Fig. S4. Consistent behavioral trends are observed across all digits: as the number of accommodated independent $T$s increases (at $\eta = 0$), image distortion progressively worsens. When the number of $T$s exceeds the network's accommodation capacity, hallucination artifacts emerge, rendering the target images unrecognizable. In contrast, the introduction of a ballistic component (e.g., $\eta = 0.01$) effectively restores target features and re-establishes recognizability.

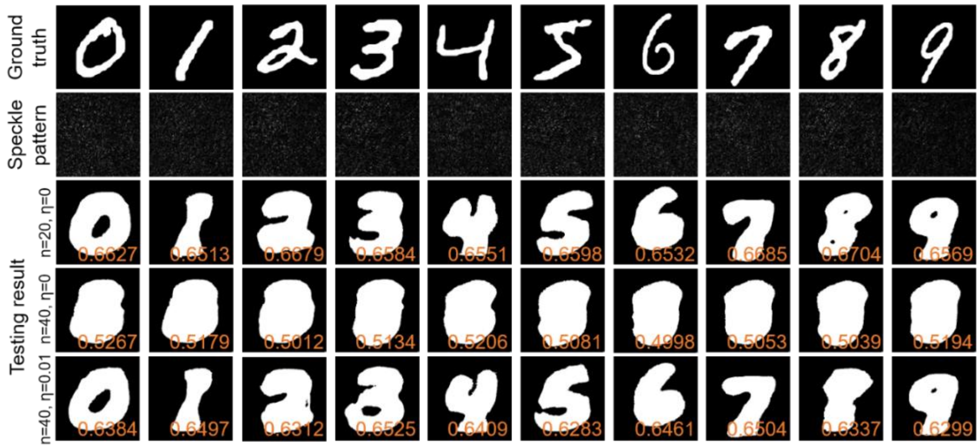


Fig. S4. Reconstruction results for different digits (0-9) with the 5-layer U-Net under controlled conditions, i.e., $n = 20$ vs. $n = 40$ without ballistic light ($\eta = 0$), and $n = 40$ without ($\eta = 0$) vs. with ($\eta = 0.01$) ballistic light. All digits exhibit consistent phenomenon: image quality degrades

with increasing $n$ in the absence of ballistic light, while the introduction of ballistic light restores feature recognition.

**Section D. Theoretical Derivation of Network Prediction under Varying Physical Conditions**

To provide a theoretical basis for network prediction performance in scattering-medium imaging, we have derived a generalized mathematical formulation applicable to a broad range of optical imaging scenarios.

1. Optimal Predictor under PCC Loss

Given the input speckle $y$ and the ground truth image $x$, let the network prediction be $\hat{x} = f(y)$. The PCC loss is typically defined as:

$$\mathcal{L}_{PCC}(f) = 1 - p(f(y), x), \tag{1}$$

where $p$ represents the Pearson Correlation Coefficient:

$$p(f(y), x) = \frac{\mathrm{Cov}(f(y), x)}{\sigma_{f(y)} \cdot \sigma_x}. \tag{2}$$

Minimizing the loss function is equivalent to maximizing the correlation coefficient $p$.

Using the law of total expectation, we establish the relationship between the covariance of $f(y)$ and $x$. For any function $f(y)$ that depends solely on $y$:

$$\mathrm{Cov}(f(y), x) = \mathrm{Cov}(f(y), E[x|y]). \tag{3}$$

Hence

$$\mathrm{Cov}(f(y), x) = E[f(y)x] - E[f(y)]E[x]. \tag{4}$$

Since

$$E[f(y)x] = E_y\left[E_{x|y}[f(y)x|y]\right] = E_y\left[f(y)E[x|y]\right], \tag{5}$$

substituting this back into Eq. (4) yields

$$\mathrm{Cov}(f(y), x) = E\left[f(y)E[x|y]\right] - E[f(y)]E\left[E[x|y]\right] = \mathrm{Cov}(f(y), E[x|y]). \tag{6}$$

Thus, the objective function becomes:

$$\max_f \frac{\mathrm{Cov}(f(y), E[x|y])}{\sigma_{f(y)} \cdot \sigma_x}. \tag{7}$$

To find the $f(y)$ that maximizes this ratio, we apply the Cauchy-Schwarz Inequality to the numerator:

$$\text{Cov}(f(y), E[x|y]) \leq \sigma_{f(y)} . \sigma_{E[x|y]}. \tag{8}$$

Substituting this back into the correlation formula, we have

$$p(f(y), x) = \frac{\text{Cov}(f(y), E[x|y])}{\sigma_{f(y)} . \sigma_x} \leq \frac{\sigma_{f(y)} . \sigma_{E[x|y]}}{\sigma_{f(y)} . \sigma_x} = \frac{\sigma_{E[x|y]}}{\sigma_x}. \tag{9}$$

The correlation coefficient $p$ reaches its upper bound (and the loss reaches its minimum) if and only if the Cauchy-Schwarz Inequality equality holds. In an inner product space, equality holds when the two vectors are linearly dependent. For the deviations of random variables, this means:

$$f(y) - E[f(y)] = \alpha\big(E[x|y] - E\big[E[x|y]\big]\big) \quad (\alpha \neq 0). \tag{10}$$

Solving for $f(y)$ gives the formula for the optimal predictor:

$$f^*(y) = \alpha E[x|y] + \beta, \tag{11}$$

where $\alpha$ and $\beta$ are arbitrary constants.

2. Optimal Predictor Model under Capacity Constraints

We assume that the image $x$ can be decomposed into three orthogonal components based on its statistical structure:

$$x = \bar{x}_c + \Delta x + \epsilon, \tag{12}$$

where $\bar{x}_c = E[x|c]$ represents the category-specific invariant; $\Delta x$ denotes the sample-specific details, satisfying $E[\Delta x|c] = 0$; and $\epsilon$ is the random noise.

When the variety of $T$ exceeds the network capacity and becomes unresolvable, the network suffers from observational ambiguity regarding the specific operator $T_i$. In this regime, the optimal predictor $f^*(y)$ under PCC loss tends toward:

$$f^*(y) \approx \alpha\left(\sum_c P(c|y)\bar{x}_c + E[\Delta x|y]\right) + \beta. \tag{13}$$

2.1 With Residual Ballistic Light ($\text{Var}(\bar{x}_c) \gg 0$)

Due to the overloading of $T$, it becomes impossible to recover the sample-specific details $\Delta x$ from the observation $y$, leading to $E[\Delta x|y] \to 0$. However, the network can still identify the category

through the statistical properties of the speckle patterns, meaning that the posterior probability $P(c|y)$ maintains high confidence. Predictor behavior:

$$f^*(y) \to \alpha\bar{x}_c + \beta. \tag{14}$$

The network outputs the categorical mean template (e.g., the average shape of the digit "5"). In this regime, $\bar{x}_c$ is captured by the network as a "statistical invariant".

2.2 Without Ballistic Light ($\mathrm{Var}(\bar{x}_c) \approx 0$)

In this regime, $\bar{x}_c = E[x|c] \approx E[x] = \mu_{global}$ (the global mean). Simultaneously, as $T$ becomes unresolvable, the sample-specific details $\Delta x$ are completely smoothed out in the $y$ space. Predictor behavior:

$$f^*(y) \to \alpha\mu_{global} + \beta. \tag{15}$$

The network outputs a featureless global-mean gray image.

**Section E. Sanity Analysis of the Artifact-Density Threshold**

We assessed the sensitivity of the artifact density metric by evaluating it over a range of threshold values near the selected baseline of 0.5. The results, presented in Fig. S5, demonstrate that the relative trends of artifact density (i.e., the relative difference across different experimental groups) remain consistent despite minor threshold adjustments. This outcome validates the rationality and robustness of the selected threshold for quantitative evaluation.

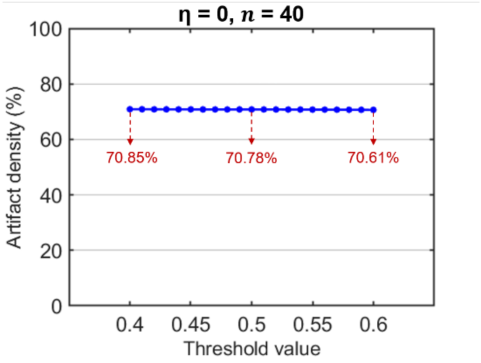


Fig. S5 Sanity check for the artifact density metric. The calculated artifact density is evaluated over a small range of threshold values near the original setting of 0.5. The consistent trend across threshold adjustments validates the reliability of the chosen metric.
