# Supplementary material for: Physical mechanisms governing generalization and hallucination in deep learning for imaging through scattering media
Source: Nat Commun. 2026 Apr 23;17:5616. doi: 10.1038/s41467-026-72304-z (PMC13315912; doi:10.1038/s41467-026-72304-z)
Supplement: Supplementary file 2 — Transparent Peer Review file [file 41467_2026_72304_MOESM2_ESM.pdf]

nature portfolio

Peer Review File

Physical Mechanisms Governing Generalization and Hallucination in Deep Learning for Imaging through Scattering Media

Corresponding Author: Professor Honglin Liu

Parts of this Peer Review File have been redacted as indicated to remove third-party material.

This file contains all reviewer reports in order by version, followed by all author rebuttals in order by version.

Version 0:

Reviewer comments:

Reviewer #1

(Remarks to the Author)
In this manuscript, the authors propose a physical study of the limitations of neural network models designed to reconstruct data acquired through scattering media. The goal of this work is to explore the physical mechanisms responsible for a model's generalization and hallucination behavior. Overall, the paper is well written, and the authors are clear about their goals. Some of the findings of this work, such as the impact of residual ballistic light over scattered light in the model's performance, pose a valuable contribution to the field of computational and scattering optics. However, I find that the scope of study is quite narrow, and I encourage the authors to explore the topic deeper to expand the impact and applicability of this work. Below I describe some of my perceived limitations of this work that I believe are addressable and can fall within the scope of this paper.

1. As motivation for their work, the authors describe the limitations that neural network models face in real-world scenarios. They present their study as an attempt to explain the physical constraints of these models, with the goal of making the findings applicable to practical contexts. They then mention contexts in which this work could be applicable (i.e., navigation in fog or underwater imaging). However, in the discussion and conclusion of the paper there is no mention how this work could be extrapolated to these areas, which are significantly less controllable than the experimental setup presented. It would benefit both the authors and the readers to include a detailed discussion on how this could be extrapolated and employed to define the physical limits of models in other contexts.
a. In addition to this, I would like to point out that, according to the results of this work, the scattering maps are never not overlapping (as the mask is 5 mm and the maximum displacement is 4.76 mm). In addition, the results demonstrate that the model breaks quickly with insufficient overlap. This should be included in the discussion of the applicability and extrapolation of this work.
2. In the methods section, the authors mention they generate the scattering patterns using the MNIST dataset, as we see in all figures. I have some concerns regarding the dataset:
a. Digit 5 is the only one shown in the results section. I would like to clarify whether this was the only digit present in the dataset, or if it is shown because it best makes the point the authors are tying to make. If the latter is true, I recommend including supplementary examples of the performance of other digits.
b. For each T in training, 5000 speckle target pairs are collected. Are these targets the same for all Ts? Were these also the targets used for testing? If this is the case, I would like for the authors to clarify it and justify this choice. How do the authors expect the model to perform if more training and testing target data variability is introduced?
3. The study of the impact of residual ballistic light is a big focus of this work, understandably so. This study effectively isolates the networks focus and ability to extract information from different signals.
a. Once the impact of ballistic light is assessed in figure 3, what is the motivation to continue including it in the study and further results? Since the simulations without residual ballistic signal are not applicable experimentally, what is the purpose of continuing to study the models' prediction abilities in these scenarios?
b. The authors should consider studying the effects of the diffuser on this physical limitation. At what physical characteristics will a scattering medium generate residual ballistic-free data, if any?
4. The manuscript is focused on identifying the interdependence limits of different training and testing Ts to define the

generalization limits of the network. I believe the way this concept is presented in the work is a bit convoluted, as it includes multiple terms such as mapping translation (∂x, ∂y) and number of maps (n). I believe the authors could consider using a different terminology, such as % overlap, to better define these mappings. This would make the text easier to follow for the reader, when trying to understand the different experimental setups.
5. In the introduction, the authors mention approaches that are regularly employed in the field to mitigate the limits of deep learning models, such as creative training schemes or data augmentation. I would be interested in seeing whether the results of this work would be affected by training with the GAN, or even CyleGAN, approach, or by augmenting the data (as I mention in point 3).

(Remarks on code availability)
no code

Reviewer #2

(Remarks to the Author)
In this work, the authors present an experimental study on the generalization capability and hallucination effect of neural networks for imaging through scattering media. By training a network to reconstruct handwritten digits from speckle images, the authors demonstrate that hallucinations arise when the network's generalization capacity is exceeded. The reviewer appreciates the efforts of the authors to address the issues of generalization and hallucination, which are long-standing challenges in deep learning. However, the contribution of the presented work has not been clearly demonstrated or sufficiently supported by solid evidence, making it difficult to assess its novelty and broader impact beyond experimentally confirming certain widely recognized notions in deep learning through a specific case study on imaging through scattering.The revision suggestions are as following.

1. A primary concern lies in the definition and interpretation of model generalization. Specifically, when solving inverse problems in the form of Y=TX as described in the manuscript, generalization may refer to adaptability across both the mapping functions T and the data distributions X. This work solely focuses on evaluating the mapping function T, which may diverge from the common understanding of generalization in the deep learning community. If the study aims to address more general deep learning issues, quantitative evaluations of dataset generalization should be included. Otherwise, the authors should explicitly clarify the scope of this work and its intended range of applications.

2. The authors claim that one of the main contributions of this work is that it "resolves long-standing debates about whether hallucinations stem from data imperfections or model limitations". While the presented results suggest that hallucinations can emerge when the network's capacity on different physical settings is exceeded, no quantitative analysis is provided on the role of data imperfections. To support this claim, the authors should conduct ablation experiments and evaluate network performance when trained on non-ideal or imperfect datasets.

3. For the specific coherent imaging model adopted in this work, the reviewer notes that the relationship between the input image X and output speckle intensity Y is nonlinear. The mapping function T that maps X to Y is a nonlinear operator which cannot be represented as a matrix. The use of matrix notation throughout the manuscript may therefore be misleading and should be revised for consistency with the underlying physics.

4. Although the authors provide detailed quantitative studies on network generalization in imaging through scattering, it remains unclear how the broader research community can practically benefit from these results. For example, are there any general principles to follow when deriving the capacity of the mapping function T in other imaging applications? The authors also mentioned possible relevance to natural language processing, but the connection between the current findings and such applications should be further clarified or supported.

5. The authors studied the generalizability of the network to different mapping T, which is constructed by laterally translating the diffuser. But it seems that translating the diffuser only spans a subspace of T. The authors should discuss how other experimental parameters (e.g., the object-to-diffuser and diffuser-to-sensor distances, diffuser feature sizes and statistics) affect the generalizability.

6. It is recommended that the authors include a discussion or ablation study on whether the choice of network architecture affects the generalization capability and the conclusions drawn from this work.

7. The authors are expected to thoroughly proofread and polish the language and expressions during the revision. Improvements can be made, for example:
- "reconstructed images at n = 31, 36, 41, and 51 fail to construct the images." (Caption of Fig. 3) this statement should be clarified for meaning and consistency.
- "In the above results all testing matrices are seen or partially seen during training," should end with a period.

(Remarks on code availability)

Reviewer #3

(Remarks to the Author)

The manuscript investigates why deep networks generalize poorly across changing scattering conditions and why "hallucinations" emerge. Using diffuser shifts to induce different forward mappings and a U-Net to reconstruct MNIST digits from speckle intensities, the authors argue: (1) a single network's generalization is bounded by how many distinct inverse mappings it can encode; (2) residual ballistic light stabilizes predictions across mapping changes; and (3) when mapping diversity exceeds capacity, hallucinations rise. The core hypothesis is interesting and potentially useful, but the current evidence over-interprets intensity-only measurements as "TM variation" and attributes stabilization specifically to ballistic light without isolating that factor.

Here are my major concerns:

1.Transmission matrix the author claim is not the real transmission matrix in scattering model or linear imaging system. Throughout the paper, T is treated like a transmission matrix in the wavefront-shaping sense, but the setup measures intensity only under only one fixed-angle coherent illumination without interferometric reference or phase diversity. In other words, the work varies unknown forward mappings by shifting the diffuser and infers independence from intensity cross-correlation. The actual transmission matrix from a physical perspective is not calibrated for each position. The author cannot claim that the transmission matrix is independent or not in this case. Please align the wording accordingly ("forward mapping indexed by diffuser shift" rather than "transmission matrix"), and temper numerical claims that depend on counting "independent T's."

2. Ballistic-light ablation is confounded with scattering strength/statistics.
The paper argues that ~1% ballistic residue anchors generalization. In simulation, that fraction is changed by altering the phase-screen statistics. So the "β knob" also changes the scattering distribution. That makes β=0 vs β=0.01 an informative but confounded comparison. In experiment, the ~1% figure comes from the nonzero tail of a correlation-vs-shift curve, which is an indirect proxy. I'd suggest: (i) add a small coaxial bypass/reference path so you can vary an invariant component without changing the diffuser; (ii) do the software analogue where you hold the scattered field statistics fixed and just add an invariant term; and (iii) run a DC/high-pass removal test to show that taking out the coherent background erases the "anchor" effect. Any one of these would make the causal story much stronger.

3.Independence and leakage.
Some test regions partially overlap trained areas, and "independence" is defined via intensity correlation only. Which is not convincing. To rule out leakage and memory-effect coupling, stricter non-overlap in space and a quantitative operator-distance metric are needed.

4. "Hallucination/artifact density" needs a precise definition and a sanity check

5. Test cases appear to have different geometric overlap with the training regions("Test 1 coincided with S_0; Tests 2–4 were shifted by 10/20/50 µm," and there is partial overlap because the illuminated area is a large circle). Mixing test sets with unequal train–test overlap on the same PCC-vs-n plot(eg.fig3) makes the curves hard to interpret: the metric then conflates (i) the number of mappings n and (ii) the effective leakage/overlap between training and test fields of view. Please separate these factors.

Minor:
In the Introduction, "generation" appears to be a typo for "generalization" when describing robustness to unseen conditions; Please correct this and conduct a thorough proofreading of the manuscript for similar issues.

(Remarks on code availability)

Version 1:

Reviewer comments:

Reviewer #2

(Remarks to the Author)
The revised manuscript has fully considered the comments of the reviewers. Current version highlights the significance of the proposal and the methodology is well presented.

It is suggested to explain the meaning of ccc in Figure 1c.
It is suggested to mark the speckle pattern and its direction in Figure 2a.
It is suggested to check whether N_bg is N_gt in the sentence 'N_bg is the total pixel count in Y_gt', below Figure 7.

(Remarks on code availability)

Reviewer #3

(Remarks to the Author)
The authors have addressed the major technical and conceptual concerns. The revised manuscript clarifies the physical interpretation of the learned inverse mapping. The discussion of ballistic and broadband components has been refined, and the experimental limitations are now more explicitly addressed.

(Remarks on code availability)
The code on GitHub has detailed README file with detailed instructions for installing and running.
(https://github.com/XuyuZhang98/Code-for-imaging-through-scattering-media-based-on-deep-learning.git)

The authors also provided a Code Ocean capsule; however, it appears to be set to private, preventing reviewer-accessible execution.

Open Access This Peer Review File is licensed under a Creative Commons Attribution 4.0 International License, which permits use, sharing, adaptation, distribution and reproduction in any medium or format, as long as you give appropriate credit to the original author(s) and the source, provide a link to the Creative Commons license, and indicate if changes were made.
In cases where reviewers are anonymous, credit should be given to 'Anonymous Referee' and the source.
The images or other third party material in this Peer Review File are included in the article’s Creative Commons license, unless indicated otherwise in a credit line to the material. If material is not included in the article’s Creative Commons license and your intended use is not permitted by statutory regulation or exceeds the permitted use, you will need to obtain permission directly from the copyright holder.
To view a copy of this license, visit https://creativecommons.org/licenses/by/4.0/

Title: *From Disorder to Design: Physical Mechanisms Governing Generalization and Hallucination in Deep Learning for Imaging Through Scattering Media*

Journal: *Nature Communications*

Response to Reviewers:

We are grateful to the reviewers for their time and the constructive comments on our manuscript. We have found the feedback to be highly insightful, helping us to identify ambiguities and significantly improve the quality and scope of our work.We have thoroughly revised the manuscript to address all concerns raised. New experiments and theoretical derivations have been added to bolster our conclusions. Below, we provide a point-by-point response to the specific comments. All major revisions are highlighted in red in the revised manuscript.

Reviewer #1 (Remarks to the Author):

In this manuscript, the authors propose a physical study of the limitations of neural network models designed to reconstruct data acquired through scattering media. The goal of this work is to explore the physical mechanisms responsible for a model's generalization and hallucination behavior. Overall, the paper is well written, and the authors are clear about their goals. Some of the findings of this work, such as the impact of residual ballistic light over scattered light in the model's performance, pose a valuable contribution to the field of computational and scattering optics. However, I find that the scope of study is quite narrow, and I encourage the authors to explore the topic deeper to expand the impact and applicability of this work. Below I describe some of my perceived limitations of this work that I believe are addressable and can fall within the scope of this paper.

Response: We sincerely thank the reviewer for their positive feedback and constructive suggestions. We have carefully revised the manuscript to address the points raised, and all major revisions are highlighted in red in the updated version. Please see our detailed responses below.

Comment #1. As motivation for their work, the authors describe the limitations that neural network models face in real-world scenarios. They present their study as an attempt to explain the physical constraints of these models, with the goal of making the findings applicable to practical contexts. They then mention contexts in which this work could be applicable (i.e., navigation in fog or underwater imaging). However, in the discussion and conclusion of the paper there is no mention how this work could be extrapolated to these areas, which are significantly less controllable than the experimental setup presented. It would benefit both the authors and the readers to include a detailed discussion on how this could be extrapolated and employed to define the physical limits of models in other contexts.

a. In addition to this, I would like to point out that, according to the results of this work, the scattering maps are never not overlapping (as the mask is 5 mm and the maximum displacement is 4.76 mm). In addition, the results demonstrate that the model breaks quickly with insufficient overlap. This should be included in the discussion of the applicability and extrapolation of this work.

Response: Thanks for the valuable suggestions. We agree that extending controlled experiments to real-world scenarios is crucial for demonstrating the broader impact of out study. This work elucidates a core mechanism: **hallucination occurrence when system diversity exceeds network capacity**. This principle applies universally to all dynamic scattering environments such as dense fog and seawater. Beyond guiding network architecture design (e.g., developing hybrid networks trained on both ballistic and scattered light), our framework may inform training data collection (e.g., determining the number of training samples with varying states to maintain generalization) and assess performance limits across different application scenarios.

Regarding the "overlapping" issue, we clarify that overlapping illumination areas do not necessarily imply correlation of scattering maps. Each point in the scattering pattern results from the coherent superposition of fields from all points on the diffuser. Beyond the isoplanatic range (40 μm in this work), two scattering maps (with $\eta = 0$) become uncorrelated. Further direct evidence is provided by the observation that a network trained one one map fails to generate to another—even with significant spatial overlap (see Row 2, labeled T_1, Column $\Delta y = 50\ \mu m$ in Fig. 6a of the revised manuscript).

This principle is aligned with our prior study [Photonics Research 11(6): 1038 (2023), i.e., Ref. 28 in the manuscript], where non-overlapping scattering maps yielded analogous results (Fig. 3c in the captioned reference). Although the illumination areas (5 mm in diameter) overlap significantly during a 40 μm step shift, the resulting scattering maps and corresponding transmission matrices (TMs) remain independent (i.e., uncorrelated) due to the coherent superposition of fields and the random nature of the scattering medium.

We have expanded the discussions on these points in the revised manuscript on Pages 5, 6, 15, and 16.

Comment #2. In the methods section, the authors mention they generate the scattering patterns using the MNIST dataset, as we see in all figures. I have some concerns regarding the dataset:
a. Digit 5 is the only one shown in the results section. I would like to clarify whether this was the only digit present in the dataset, or if it is shown because it best makes the point the authors are trying to make. If the latter is true, I recommend including supplementary examples of the performance of other digits.
b. For each T in training, 5000 speckle target pairs are collected. Are these targets the same for all Ts? Were these also the targets used for testing? If this is the case, I would like for the authors to clarify it and justify this choice. How do the authors expect the model to perform if more training and testing target data variability is introduced?

Response: We apologize for any confusion regarding the target selection. The handwritten digits '5' was chosen for visual continuity and comparability across results. To address this concern, we have added reconstruction results for all digits (0-9) in the Supplementary Information (Section C, Fig. S4), which is also shown below (Fig. R1) for your information. As seen, these results consistently exhibit the same trends.

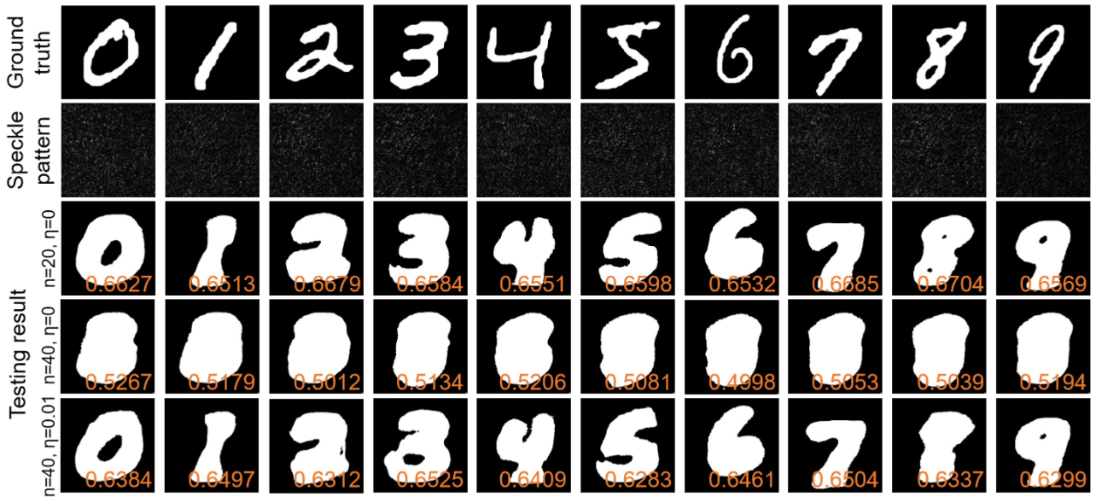

Fig. R1. Reconstruction results for different digits (0–9) with the 5-layer U-Net under controlled conditions: $n = 20$ vs. $n = 40$ without ballistic light ($\eta = 0$), and $n = 40$ without ($\eta = 0$) vs. with ($\eta = 0.01$) ballistic light. All digits exhibit a consistent phenomenon: image quality degrades with increasing n in the absence of ballistic light, while the introduction of ballistic light restores feature recognition.

Regarding the datset, the standard MNIST dataset was split into two subsets, i.e., images #1-50,000 for training and #50,001-60,000 for testing. For each transmission matrix T in training, 5,000 target images were randomly selected from the training subset, and corresponding speckle patterns were collected. Testing targets were drawn exclusively from the testing subset and were never seen during training. We found that the network performance tends to stabilize when the number of training pairs reaches ~3,000 per T. We believe that increased variability in training and testing targets would not alter the core conclusions.

We have added the results of different digits as suggested and clarified these points in the manuscript (Page 6) and in the Supplementary Information (Section C).

Comment #3. The study of the impact of residual ballistic light is a big focus of this work, understandably so. This study effectively isolates the networks focus and ability to extract information from different signals.

a) Once the impact of ballistic light is assessed in figure 3, what is the motivation to continue including it in the study and further results? Since the simulations without residual ballistic signal are not applicable experimentally, what is the purpose of continuing to study the models' prediction abilities in these scenarios?

b) The authors should consider studying the effects of the diffuser on this physical limitation. At what physical characteristics will a scattering medium generate residual ballistic-free data, if any?

Response: Thanks for the questions stemming from thorough deliberation.

a) While residual ballistic light is important, it is not the sole focus. Our study also investigates the cause of poor generalization under dynamic conditions and the emergence of hallucinatory artifacts. If we stopped at Fig. 3, one might question whether generalization limits truly stem from the network's finite capability to accommodate distinct inverse mappings ($T^{-1}s$); while

hallucinations arise when this capacity is exceeded, resulting in unconstrained, non-physical predictions, the impact of residue ballistic light has yet to be widely and profoundly grasped. Presenting comparative results with and without ballistic light clarifies the underlying physical principles. Currently, in most imaging techniques through scattering media, the role of ballistic light cannot be neglected, although it is often not recognized. Understanding its contribution helps determine when and how to leverage different light components for improved imaging.

For a comprehensive understanding, we derived the prediction:

1. Optimal predictor under PCC loss

Given the input speckle y and the ground truth image x, let the network prediction be $\hat{x} = f(y)$. The PCC loss is typically defined as:

$$\mathcal{L}_{PCC}(f) = 1 - p(f(y), x), \quad (1)$$

where p represents the Pearson Correlation Coefficient:

$$p(f(y), x) = \frac{\text{Cov}(f(y), x)}{\sigma_{f(y)} \cdot \sigma_x}. \quad (2)$$

Minimizing the loss function is equivalent to maximizing the correlation coefficient p.

Using the law of total expectation, we establish the relationship between the covariance of $f(y)$ and x. For any function $f(y)$ that depends solely on y:

$$\text{Cov}(f(y), x) = \text{Cov}(f(y), E[x|y]). \quad (3)$$

Hence

$$\text{Cov}(f(y), x) = E[f(y)x] - E[f(y)]E[x]. \quad (4)$$

Since

$$E[f(y)x] = E_y\left[E_{x|y}[f(y)x|y]\right] = E_y\big[f(y)E[x|y]\big], \quad (5)$$

substituting this back into Eq. (4) yields

$$\text{Cov}(f(y), x) = E\big[f(y)E[x|y]\big] - E[f(y)]E\big[E[x|y]\big] = \text{Cov}(f(y), E[x|y]). \quad (6)$$

Thus, the objective function becomes:

$$\max_f \frac{\text{Cov}(f(y), E[x|y])}{\sigma_{f(y)} \cdot \sigma_x}. \quad (7)$$

To find $f(y)$ that maximizes this ratio, we apply the Cauchy-Schwarz Inequality to the numerator:

$$\text{Cov}(f(y), E[x|y]) \leq \sigma_{f(y)} \cdot \sigma_{E[x|y]}. \quad (8)$$

Substituting this back into the correlation formula, we have

$$p(f(y), x) = \frac{\text{Cov}(f(y), E[x|y])}{\sigma_{f(y)} \cdot \sigma_x} \leq \frac{\sigma_{f(y)} \cdot \sigma_{E[x|y]}}{\sigma_{f(y)} \cdot \sigma_x} = \frac{\sigma_{E[x|y]}}{\sigma_x}. \quad (9)$$

The correlation coefficient p reaches its upper bound (and the loss reaches its minimum) if and only if the Cauchy-Schwarz Inequality equality holds. In an inner product space, equality holds when the two vectors are linearly dependent. For the deviations of random variables, this means:

$$f(y) - E[f(y)] = \alpha\big(E[x|y] - E\big[E[x|y]\big]\big) \quad (\alpha \neq 0). \quad (10)$$

Solving for $f(y)$ gives the formula for the optimal predictor:

$$f^*(y) = \alpha E[x|y] + \beta, \quad (11)$$

where α and β are arbitrary constants.

2. Optimal predictor model under capacity constraints

Assume that the image x can be decomposed into three orthogonal components based on its statistical structure:

$$x = \bar{x}_c + \Delta x + \epsilon, \tag{12}$$

Where $\bar{x}_c = E[x|c]$ represents the category-specific invariant; Δx denotes the sample-specific details, satisfying $E[\Delta x|c] = 0$; and ϵ is the random noise.

When the variety of T exceeds the network capacity and becomes unresolvable, the network suffers from observational ambiguity regarding the specific operator T_i. In this regime, the optimal predictor $f^*(y)$ under PCC loss tends toward:

$$f^*(y) \approx \alpha \left(\sum_c P(c|y)\bar{x}_c + E[\Delta x|y] \right) + \beta. \tag{13}$$

2.1 Residue ballistic light ($\mathrm{Var}(\bar{x}_c) \gg 0$)

Due to the overloading of T, it becomes impossible to recover the sample-specific details Δx from the observation y, leading to $E[\Delta x|y] \to 0$. However, the network can still identify the category through the statistical properties of the speckle patterns, meaning that the posterior probability $P(c|y)$ maintains high confidence. Predictor behavior:

$$f^*(y) \to \alpha \bar{x}_c + \beta. \tag{14}$$

The network outputs the categorical mean template (e.g., the average shape of the digit “5”). In this regime, $\bar{x}_c$ is captured by the network as a “statistical invariant”.

2.2 Without ballistic light ($\mathrm{Var}(\bar{x}_c) \approx 0$)

In this regime, $\bar{x}_c = E[x|c] \approx E[x] = \mu_{global}$ (the global mean). Simultaneously, as T becomes unresolvable, the sample-specific details Δx are completely smoothed out in the y space. Predictor behavior:

$$f^*(y) \to \alpha \mu_{global} + \beta. \tag{15}$$

The network outputs a featureless global-mean gray image.

b) For different diffusers, the isoplanatic ranges and ballistic proportions η may vary. However, the fundamental principles we identify remain general. Please refer to Photonics Research 11(6): 1038 (2023) (Ref. [28] in the manucript) for a systematic analysis on the impact of η at different isoplanatic ranges. Basically, a volumetric scattering medium can be considered ballistic-free when its optical thickness is sufficiently large, though the exact threshold depends on the scenario. Phase plates fabricated with lithography or coarse-grit diffusers, such as Edmund Optics, 220-grit (Optica 5, 803-813 (2018)), can also minimize ballistic residue.

We have revised the manuscript to incorporate the above information in Discussion (Page 16) in the manuscript and Section D in Supplementary Information accordingly.

Comment #4. The manuscript is focused on identifying the interdependence limits of different training and testing Ts to define the generalization limits of the network. I believe the way this concept is presented in the work is a bit convoluted, as it includes multiple terms such as mapping

translation (∂x,∂y) and number of maps (n). I believe the authors could consider using a different terminology, such as % overlap, to better define these mappings. This would make the text easier to follow for the reader, when trying to understand the different experimental setups.

Response: We appreciate the suggestion. However, we can hardly agree with it. When $\eta = 0$, a lateral shift beyond the isoplanatic range yields a new independent scattering map. The mapping translation and the number of maps are not convolved in this regime. Of course, for shifts smaller than the isoplanatic range (e.g., Group II in Fig. 5), some correlation exists, which we have explicitly explained. Since the isoplanatic range varies with experimental setup, using "percentage overlap" could introduce additional confusion. We have therefore retained the terms "translation" and "number of maps" but have improved the text for clarity.

Comment #5. In the introduction, the authors mention approaches that are regularly employed in the field to mitigate the limits of deep learning models, such as creative training schemes or data augmentation. I would be interested in seeing whether the results of this work would be affected by training with the GAN, or even CyleGAN, approach, or by augmenting the data (as I mention in point 3).

Response: We would like to thank the reviewer for their constructive suggestions for further research. A common hypothesis in deep learning posits that more advanced network architectures improve generalization. However, our core argument is that the observed limitation is inherently physical rather than algorithmic. Specifically, for a network designed to accommodate multiple transmission matrices (TMs), once the number of TMs exceeds the network's capacity, generalization deteriorates and hallucinations appear—a phenomenon governed by the same underlying physical principle.

Provided that the training paradigm follows the physical system from mapping relation $X = f_\theta(Y) = T^{-1}\mathcal{R}Y$, the difference between networks of varying architectures and parameter scales lies only in the number of TMs they can accommodate. As supporting evidence, please refer to the experimental results with a GAN presented in Fig. R2.

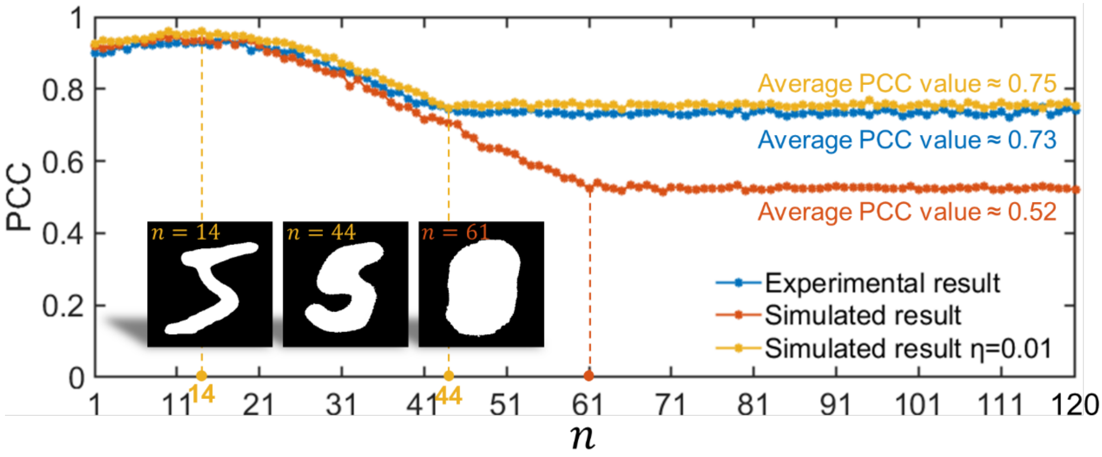

Fig. R2. Accommodation capability test of the GAN with and without ballistic light. The GAN accommodates more independent transmission matrices (T) compared to the 5-layer U-Net in the manuscript, but hallucinations similarly

emerge when *n* (here, *n* = 61) exceeds the network's capacity.

The GAN can accommodate more independent Ts. However, when the number of independent Ts exceeds its capacity (i.e., $n = 61$), the generated images also exhibit hallucinations. These findings strongly support our core conclusions.

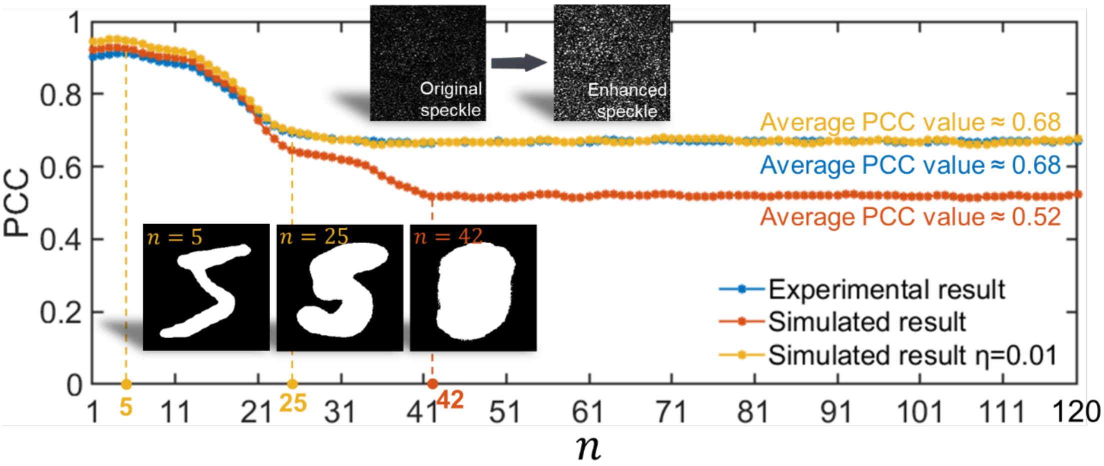

Fig. R3. Accommodation capability of the 5-layer U-Net under contrast enhancement of recorded speckle patterns.

We also tested data augmentations, e.g., contrast enhancement with the 5-layer U-Net. As shown in Fig. R3, while contrast enhancement yields a marginal improvement in image quality (evidenced by the slightly higher PCC values across all n values when compared against the referece data shown in Fig. 4), and only induces a negligible enhancement in the U-Net's capacity to accommodate different Ts, it does not alter the fundamental principle: a network has a finite capacity to accommodate independent mappings. Specifically, as the number of mappings n increases, image quality degrades progressively, distortions become increasingly severe, and hallucinations eventually occur. The results align with the conclusion of Ref. [24] that data augmentation primarily improves generalization in X, but not in T.

Reviewer #2 (Remarks to the Author):

In this work, the authors present an experimental study on the generalization capability and hallucination effect of neural networks for imaging through scattering media. By training a network to reconstruct handwritten digits from speckle images, the authors demonstrate that hallucinations arise when the network's generalization capacity is exceeded. The reviewer appreciates the efforts of the authors to address the issues of generalization and hallucination, which are long-standing challenges in deep learning. However, the contribution of the presented work has not been clearly demonstrated or sufficiently supported by solid evidence, making it difficult to assess its novelty and broader impact beyond experimentally confirming certain widely recognized notions in deep learning through a specific case study on imaging through scattering. Revision suggestions are as following.

Response: We thank the reviewer for acknowledging the value of this research and for their constructive comments, which have helped us significantly improve the manucript.

Comment #1. A primary concern lies in the definition and interpretation of model generalization. Specifically, when solving inverse problems in the form of $Y = TX$ as described in the manuscript, generalization may refer to adaptability across both the mapping functions T and the data distributions X. This work solely focuses on evaluating the mapping function T, which may diverge from the common understanding of generalization in the deep learning community. If the study aims to address more general deep learning issues, quantitative evaluations of dataset generalization should be included. Otherwise, the authors should explicitly clarify the scope of this work and its intended range of applications.

Response: We thank the reviewer for their critical and insightful comments. In deep learning, generalization encompasses both X (data) and T (system mapping) domains. To enhance generalization, techniques such as data augmentation, network architecture design, training strategies are widely adopted. However, comparing to these generalization improvements, we argue that identifying the physical roots underlying generalization limitations is of greater significance.

In Ref. [24], we showed that poor generalization in X arises when the learned mapping deviates from the true system mapping due to biased training data. By designing a speckle pattern dataset that respects spacetime homogeneity, we achieved universal generalization across diverse datasets (see Fig. R4 below; reproduced from Acta Optica Sinica, 2025, 45(19): 1911013). This demonstrates that the network learned the underlying physical mapping rather than specific image features.

[editorial note: third party material redacted]

Fig. R4 Universal generalization achieved by training with a homogeneous speckle pattern dataset. (a) Example speckle patterns from the custom-designed training dataset, which exhibits spacetime homogeneity. (b) Reconstruction results for diverse target types using the network trained solely on the speckle dataset. The network successfully recovers features from binary digits, grayscale faces, and other complex targets, demonstrating that it has learned the underlying physical system mapping rather than specific image features. Spec.: custom speckle pattern targets; Charact.: custom character targets.

In physics, T and X represent distinct factors. Deep learning can only be reliably applied in real-world scenarios if it adheres to the constraints of physical space. In this study, we focus on generalization across the T domain—what we term “physical/system generalization.”, while we also acknowledging the standard generalization definition within the broader deep learning community. We have clarified this scope in the Introduction.

Comment #2. The authors claim that one of the main contributions of this work is that it “resolves long-standing debates about whether hallucinations stem from data imperfections or model limitations”. While the presented results suggest that hallucinations can emerge when the network's capacity on different physical settings is exceeded, no quantitative analysis is provided on the role of data imperfections. To support this claim, the authors should conduct ablation experiments and evaluate network performance when trained on non-ideal or imperfect datasets.

Response: We are grateful for the insightful and thoughtful feedback from the reviewer. As demonstrated in Ref. [24], data imperfections cause the learned mapping to deviate from the true system mapping, leading to generalization deficiency in X, prediction distortions, and even failures (i.e., hallucinations). Meanwhile, neural networks have a finite capacity to accommodate distinct T s, which constitutes a fundamental model limitation. When this capacity is exceeded, generalization degrades and hallucinations emerge. Together, these two factors resolve the long-standing debates in the field regarding whether hallucinations arise from data imperfections or model limitations. We have refined this argument in the manuscript (Paragraph 1 in Discussion).

Comment #3. For the specific coherent imaging model adopted in this work, the reviewer notes that the relationship between the input image X and output speckle intensity Y is nonlinear. The mapping function T that maps X to Y is a nonlinear operator which cannot be represented as a matrix. The use of matrix notation throughout the manuscript may therefore be misleading and should be revised for consistency with the underlying physics.

Response: Thanks for pointing out the issue. The relationship between the input image X and output speckle intensity Y, $Y =| TX |^2$, is indeed nonlinear. The retrieval process involves $X = T^{-1}\mathcal{R}Y$, where $\mathcal{R}$ is an intensity-to-field transformation. A neural network learns both the linear mapping T^{-1} and the non-linear transformation $\mathcal{R}$. Since $\mathcal{R}$ is a constant across different Ts, we simplify the notation for clarity. We have revised the manuscript to explicitly clarify this point in the "Mathematical Formulation of Speckle Pattern Retrieval in Deep Learning" section of the Methods.

For better understanding of $X = T^{-1}\mathcal{R}Y$, please see the following results (included in a new version of Ref. [24] that is currently under review):

a) Training with a speckle dataset (space homogeneity + grayscale tuning) enables universal generalization (Fig. R5).

b) Training with a binary grating dataset (space homogeneity only) leads to limited generalization, as the network cannot learn the grayscale fitting curve (Fig. R6).

c) When trained on complex optical field instead of intensity, the binary grating dataset does enable generalization to grayscale images (Fig. R7), as there is no requirement on nonlinear intensity-to-field transformation. Together with a), the results indicate that the network can simultaneously learn the inverse mapping T^{-1} and the necessary transformations, e.g., the intensity-to-field transformation $\mathcal{R}$.

[editorial note: third party material redacted]

Fig. R5 Universal generalization from a network trained on a speckle pattern dataset. The network, trained with speckle pattern pairs, successfully reconstructs both binary and grayscale targets of various features, confirming that learning from a homogeneous dataset enables universal generalization. The independent nature of speckle grains allows for a more accurate mapping to be learned, though it requires a larger number of training samples.

[editorial note: third party material redacted]

Fig. R6 Limited generalization from a network trained on a binary grating dataset. While the binary grating dataset provides spatial homogeneity, the network fails to learn the nonlinear grayscale value fitting required for the intensity

detection process ($Y = |TX|^2$). Consequently, it only achieves generalization among binary targets and cannot effectively reconstruct grayscale images.

[editorial note: third party material redacted]

Fig. R7 Universal generalization achieved by training on complex fields instead of intensities. When the network is trained using binary grating targets paired with their corresponding complex optical fields, it successfully learns the linear inverse mapping T^{-1}, enabling successful reconstruction of both binary and grayscale targets.

Comment #4. Although the authors provide detailed quantitative studies on network generalization in imaging through scattering, it remains unclear how the broader research community can practically benefit from these results. For example, are there any general principles to follow when deriving the capacity of the mapping function T in other imaging applications? The authors also mentioned possible relevance to natural language processing, but the connection between the current findings and such applications should be further clarified or supported.

Response: Thanks for the insightful comment. In this study, we focus on discussing the network's accommodation capability to mappings associated with imaging through scattering media. The core principle—**hallucination occurs when system diversity exceeds network capacity**—is general. In other imaging applications (e.g., geometric or diffraction imaging), the mappings are less complex, placing lower demands on network's learning-to-simulate capability. The same evaluation method can be extended to assess generalization capability in those contexts.

In natural language processing, models must adhere to multiple principles (e.g., linguistic ones, factual, etc). If certain principles are inadequately modeled, distortions or hallucinations will occur.

We have revised the Discussion section (Paragraph 1 & 3 on Page 16, and Paragraph 1 on Page 17) to include these connections.

Comment #5. The authors studied the generalizability of the network to different mapping T, which is constructed by laterally translating the diffuser. But it seems that translating the diffuser only spans a subspace of T. The authors should discuss how other experimental parameters (e.g., the object-to-diffuser and diffuser-to-sensor distances, diffuser feature sizes and statistics) affect the generalizability.

Response: The reviewer is correct that translating the diffuser only spans a subspace of T. Changing the object-to-diffuser or diffuser-to-sensor distances alters the field-of-view (FOV) and resolution; changing the diffuser properties affects the proportion of residue ballistic light. By using lateral translation, we isolate T-dependent effects from other variables. This approach allows us to systematically study generalization limits without confounding factors.

Comment #6. It is recommended that the authors include a discussion or ablation study on whether the choice of network architecture affects the generalization capability and the conclusions drawn from this work.

Response: Thanks for the constructive suggestion. We have conducted systematic studies on how network depth and width affect the generalization capability in X. The results will be specifically reported in another manuscript, which are also discussed herein to address the suggestion. In Fig. R8, we show how training dataset size and network architecture affect the performance. The accommodation capability of a GAN to TMs is illustrated in Fig. R9, same to the results presented in Fig. R2. Apparently, the model has its own capability to independent Ts.

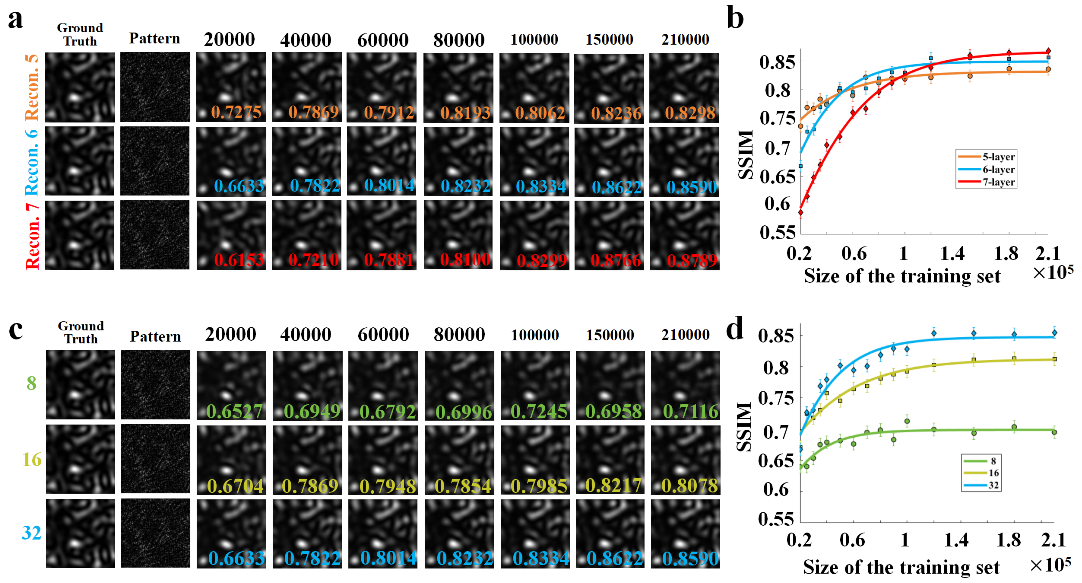

Fig. R8 Impact of training dataset size and network architecture on performance. (a) Reconstructed images from the 5-, 6-, and 7-layer U-Nets (first-layer kernels=32) trained with different dataset sizes. The SSIM value for each image is labeled below. (b) Reconstruction quality (SSIM) versus training sample size for the three networks. The curves are logistic fits, showing that deeper networks have a higher performance ceiling but require more data to reach saturation. (c) Reconstructed images from 6-layer U-Nets with different numbers of first-layer convolutional kernels (X = 8, 16, 32) under varying training sample sizes. (d) The relationship between SSIM and sample size for the 6-layer U-Nets of different widths. Wider networks show a higher potential performance but require more data for convergence.

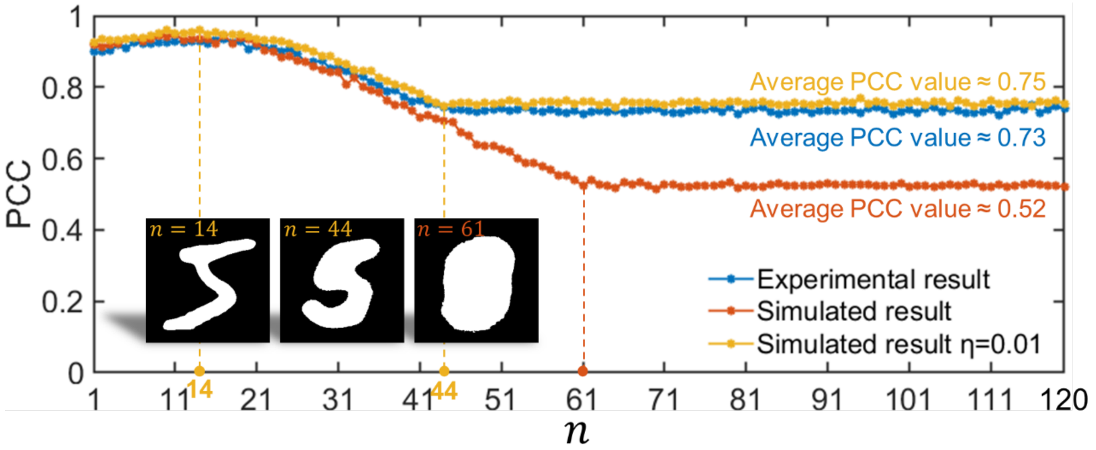

Fig. R9 Accommodation capability test of the GAN with/without ballistic light, reproduced from Fig. R2, for

comparison with other architectures.

Similarly, the accommodation capability of a 6-layer U-Net is also quantified in Fig. R10. In comparison with its 5-layer counterpart, the 6-layer architecture exhibits stronger capacity to accommodate independent Ts. However, once the number of Ts exceeds its accommodation limit, the model generates hallucinations and ultimately fails to achieve valid image reconstruction.

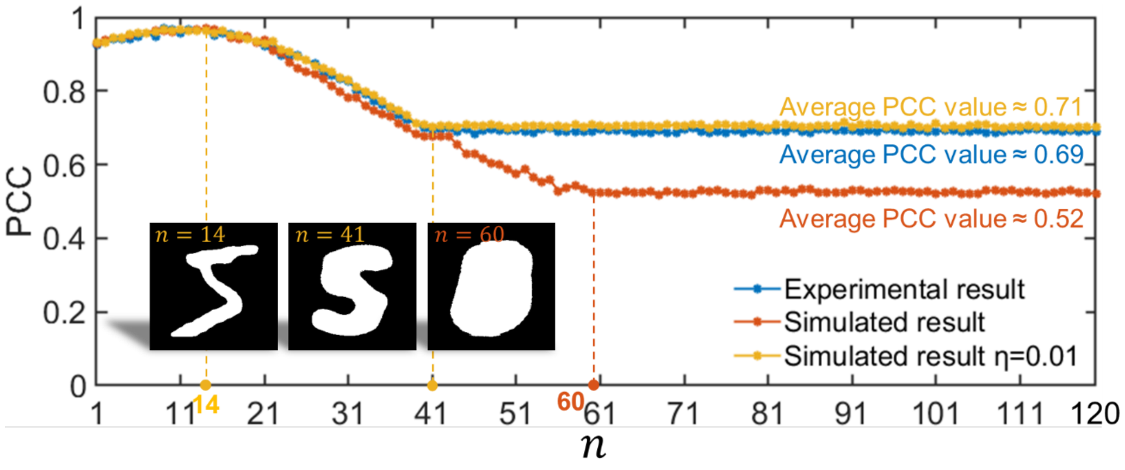

Fig. R10 Accommodation capability test of the 6-layer U-Net with and without ballistic light. Compared to the 5-layer U-Net, the deeper architecture exhibits a stronger capacity to accommodate independent T matrices, but hallucinations still emerge when its capacity is exceeded.

Overall, wider and deeper networks generally yield better performance but also require larger training datasets and longer training cycles to converge. This aligns with the widely accepted consensus. Under the same training paradigm, the generalization ability of a network (its capacity to accommodate TMs) varies with its architecture, which is associated with the network's inherent modeling capacity and structural efficiency. When exceeding the capacity limit, hallucinations will still occur. Hence, the findings of this study can be generalized beyond specific network architectures and scales. These results confirm that while architecture influences capacity, the underlying physical limitation persists. We have added this discussion in the Supplementary Information (Section A).

7. The authors are expected to thoroughly proofread and polish the language and expressions during the revision. Improvements can be made, for example:
- "reconstructed images at n = 31, 36, 41, and 51 fail to construct the images." (Caption of Fig. 3) this statement should be clarified for meaning and consistency.
- "In the above results all testing matrices are seen or partially seen during training," should end with a period.

Response: We sincerely thank the reviewer for pointing out the issue related with language and proofreading. We have conducted a full and thorough proofreading of the entire manuscript to ensure the consistency of terminology, the accuracy of expressions, and the standardization of punctuation throughout the text.

For specific examples raised above,
- About the caption of Fig. 3: the ambiguous statement “reconstructed images at n = 31, 36, 41, and 51 fail to construct the images” has been revised to “The images reconstructed at n = 31, 36, 41, and 51 fail to accurately recover the target objects” to eliminate redundancy and clarify the core meaning.
- About the sentence “In the above results all testing matrices are seen or partially seen during training”, we have added the missing period at the end and adjusted the punctuation to meet academic writing standards, resulting in “In the above results, all testing matrices are seen or partially seen during training.”

Reviewer #3 (Remarks to the Author):

The manuscript investigates why deep networks generalize poorly across changing scattering conditions and why “hallucinations” emerge. Using diffuser shifts to induce different forward mappings and a U-Net to reconstruct MNIST digits from speckle intensities, the authors argue: (1) a single network’s generalization is bounded by how many distinct inverse mappings it can encode; (2) residual ballistic light stabilizes predictions across mapping changes; and (3) when mapping diversity exceeds capacity, hallucinations rise. The core hypothesis is interesting and potentially useful, but the current evidence over-interprets intensity-only measurements as “TM variation” and attributes stabilization specifically to ballistic light without isolating that factor.

Response: We thank the reviewer for his/her insightful summary and constructive critique. Their comments precisely point out the key areas for improving the rigor of our research, which is highly valued. We have carefully addressed each point to enhance the rigor and clarity of our work. All revisions are marked in red in the updated manuscript.

Major concerns:

Comment #1. Transmission matrix the author claim is not the real transmission matrix in scattering model or linear imaging system.

Throughout the paper, T is treated like a transmission matrix in the wavefront-shaping sense, but the setup measures intensity only under only one fixed-angle coherent illumination without interferometric reference or phase diversity. In other words, the work varies unknown forward mappings by shifting the diffuser and infers independence from intensity cross-correlation. The actual transmission matrix from a physical perspective is not calibrated for each position. The author cannot claim that the transmission matrix is independent or not in this case. Please align the wording accordingly (“forward mapping indexed by diffuser shift” rather than “transmission matrix”), and temper numerical claims that depend on counting “independent T’s.”

Response: Thanks for pointing out the inaccurate expressions in the manuscript. The recorded speckle pattern is expressed as $Y = | TX |^2$, and the amplitude object can be retrieved via $X = T^{-1}\mathcal{R}Y$, where $\mathcal{R}$ denotes an intensity-to-field transformation, and T^{-1} is the inverse of the linear transmission matrix T. A neural network can simultaneously learn both the linear inverse mapping T^{-1} and the nonlinear transformation $\mathcal{R}$. Notably, $\mathcal{R}$ remains a constant across different T^{-1}s. For simplicity, the nonlinear component is neglected (for further details, please refer to our response to Comment # 3 of Reviewer # 2). We have revised the manuscript to explicitly clarify these definitions and assumptions.

Another key concern raised is that the intensity distribution measured under a single fixed-angle coherent illumination does not equate to a transmission matrix. Nevertheless, if two speckle patterns (and thus their underlying complex fields) are uncorrelated beyond the isoplanatic range, their corresponding transmission matrices are regarded independent. This can be reasoned as below.

As illustrated in Fig. R11a, when a point source illuminates a diffuser, a speckle pattern—or equivalently, a complex field—is acquired. Following a lateral shift of the diffuser, a new speckle pattern (and its corresponding complex field) is generated. Beyond the isoplanatic range (which is equivalent to the translational memory effect range), the two speckle patterns are mutually uncorrelated, and their corresponding complex fields exhibit the same uncorrelated property.

In Fig. R11b, when the point source is shifted across the object plane, within the angular memory effect range, the resulting speckle pattern (and complex field) maintains an almost identical distribution, differing only by a spatial translation. A transmission matrix is constructed by assembling the complex fields of point sources at all positions within the field-of-view (FOV). Since the complex-field components associated with different diffuser shifts are mutually uncorrelated, we can rigorously conclude that the corresponding two transmission matrices are independent. This conclusion remains valid even when the point source shift exceeds the angular memory effect range. Specially, when the object distance u is sufficiently large, the measurement setup reduces to the case of single fixed-angle coherent illumination.

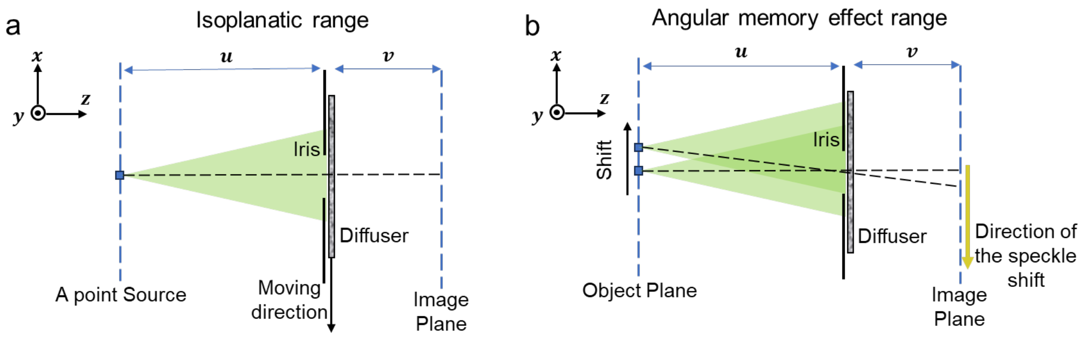

Fig. R11 Schematic illustration for establishing transmission matrix (T) independence. (a) Measuring the isoplanatic range (translational memory effect): a lateral diffuser shift beyond this range yields uncorrelated speckle patterns (and thus independent complex fields). (b) Constructing the transmission matrix: each column corresponds to the complex field from a point source within the field-of-view. Uncorrelated fields from different diffuser shifts imply independent T matrices.

We apologize for the ambiguity in our original expression. To avoid potential confusion, we have clarified this in the revised manuscript on Pages 5 & 6.

Comment #2. Ballistic-light ablation is confounded with scattering strength/statistics.

The paper argues that ~1% ballistic residue anchors generalization. In simulation, that fraction is changed by altering the phase-screen statistics. So, the “β knob” also changes the scattering distribution. That makes β=0 vs β=0.01 an informative but confounded comparison. In experiment, the ~1% figure comes from the nonzero tail of a correlation-vs-shift curve, which is an indirect

proxy. I'd suggest: (i) add a small coaxial bypass/reference path so you can vary an invariant component without changing the diffuser; (ii) do the software analogue where you hold the scattered field statistics fixed and just add an invariant term; and (iii) run a DC/high-pass removal test to show that taking out the coherent background erases the "anchor" effect. Any one of these would make the causal story much stronger.

Response: We wish to clarify that the parameter β does not appear in our manuscript. We conjecture that the parameter referenced by the reviewer is instead η. This parameter η (not β) controls the ballistic proportion.

In simulation, the ground glass diffuser is modeled as a random phase screen expressed by $\exp\left[-jC\phi(x,y)\right]$, where the phase distribution $\phi(x,y)$ follows a Gaussian distribution. The proportion of the ballistic component (η) is regulated by adjusting the parameter C, and the quantitative relationship between C and η is calibrated beforehand. For each value of C, we obtained the normalized cross-correlation coefficient curve (i.e., the correlation-vs.-shift curve), as illustrated in the corresponding figure reproduced from Ref. 28. We selected the specific value of C for which the tail of this curve matches the tail value derived from experimental measurements.

Clearly, the residue ballistic light proportions in both experiments and simulations were quantified using the identical method and consistent evaluation criteria. Furthermore, our approach to controlling η in simulations is consistent with Method ii proposed by the reviewer.

Regarding the suggested Method i, we would like to emphasize that achieving precise control and quantitative analysis of ballistic and scattered light poses substantial experimental challenges. Even when a bypass is incorporated into the setup, quantitative characterization remains unfeasible in practice, mainly due to the detrimental effects of experimental instability and noise interference.

As for Method iii, we wish to further clarify that the ballistic component inherently contains a broad range of spatial frequency components after encoding the target related information. This fundamental property makes it challenging to differentiate the ballistic component from the scattered counterpart.

[editorial note: third party material redacted]

Fig. R12 Calibration of ballistic light proportion (η) from Ref. 28. The tail value of the normalized cross-correlation coefficient curve (correlation vs. shift) is used to quantify the residual ballistic component. The simulation parameter C is tuned to match the experimental η value.

Comment #3. Independence and leakage.
Some test regions partially overlap trained areas, and "independence" is defined via intensity correlation only. Which is not convincing. To rule out leakage and memory-effect coupling, stricter non-overlap in space and a quantitative operator-distance metric are needed.

Response: We acknowledge the reviewer's observation that partial overlap exists between certain

test regions and trained areas. To address the concern regarding mapping independence, we emphasize the following key points:

First, the independence of forward mappings is equivalent to the independence of transmission matrices (TMs)—a relationship we have elaborated in detail in our response to Comment #1 of Reviewer # 3, and we kindly refer the reviewer to that section for comprehensive reasoning.

Second, experimentally, a network trained on one TM fails completely on another TM displaced beyond the isoplanatic range, confirming no information leakage, and partial overlap in spatial regions does not compromise the independence of the underlying mappings.

Our prior study (Ref. 28) used non-overlapping maps and reported consistent phenomena. For example, results presented in Fig. 6a (previous Fig. 5a) of revised manuscript align with the patterns illustrated in Fig. 3c of Ref. 28, further validating the reliability of our approach. We have strengthened this argument in the revised manuscript (Pages 5 & 6).

Comment #4. “Hallucination/artifact density” needs a precise definition and a sanity check.

Response: Thanks for this valuable suggestion. We have clarified the definition of artifact density (referenced in Fig. 7) in our manuscript: it refers to the percentage of bright pixels in the reconstructed image that deviate from the ground truth. The formal mathematical expression of this metric is given below:

$$D_{artifact} = \frac{\sum_{(i,j)\in \mathrm{N}_{gt}} I\left(\left|Y_{pred}(i,j)-Y_{gt}(i,j)\right|\geq 0.5\right)}{\sum_{(i,j)\in \mathrm{N}_{gt}} I\left(\left|Y_{gt}(i,j)\right|\geq 0.5\right)} \times 100\%.$$

In this formula, Y_{pred} and Y_{gt} are binarized reconstruction image and ground-truth image, respectively; N_{bg} is the total pixel count in Y_{gt}; $I(\cdot)$ is an indicator function, which takes a value of 1 when the internal condition holds, and 0 otherwise. The corresponding modifications can be found on Page 14, Paragraph 3.

Regarding the sanity check suggested by the reviewer, we carried out a threshold sensitivity test by evaluating artifact density using a small range of threshold values near the original setting of 0.5. The results in Fig. R13 demonstrate that the trend of artifact density (i.e., the relative difference across different experimental groups) remains consistent regardless of minor threshold adjustments. This outcome validates the rationality of the selected threshold and the reliability of the artifact density metric for quantitative evaluation.

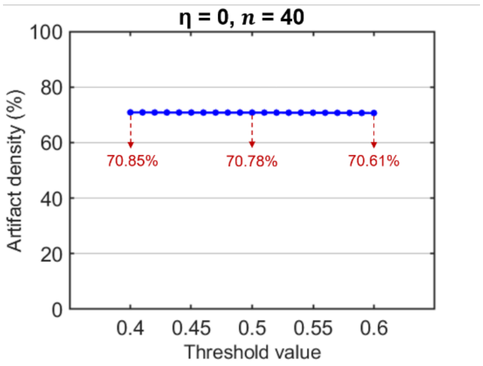

Fig. R13 Sanity check for the artifact density metric. The calculated artifact density is evaluated over a small range of threshold values near the original setting of 0.5. The consistent trend across threshold adjustments validates the reliability and robustness of the chosen metric for quantitative evaluation.

Comment #5. Test cases appear to have different geometric overlap with the training regions ("Test 1 coincided with S_0; Tests 2–4 were shifted by 10/20/50 μm," and there is partial overlap because the illuminated area is a large circle). Mixing test sets with unequal train–test overlap on the same PCC-vs-n plot (e.g. fig. 3) makes the curves hard to interpret: the metric then conflates (i) the number of mappings n and (ii) the effective leakage/overlap between training and test fields of view. Please separate these factors.

Response: It is correct that Test 1 coincides with the reference position S_0, whereas Tests 2–4 are offset by 10, 20, and 50 μm, respectively. That said, Fig. 3 exclusively employs Test 1 data under varying n values to evaluate the network's accommodation capacity of different Ts. The decay of PCC is purely caused by the increasement of n, rather than the diminished overlap between the test and reference areas. To address this potential confusion, we have update the caption of Fig. 4 in revision (Fig. 3 in the original manuscript).

Minor

In the Introduction, "generation" appears to be a typo for "generalization" when describing robustness to unseen conditions; Please correct this and conduct a thorough proofreading of the manuscript for similar issues.

Response: Thank you for the meticulous comment. We confirm that "generation" was indeed a typo for "generalization" in the first paragraph in Introduction section, and we have corrected this error accordingly. We have also performed a thorough proofread of the entire manuscript, including the texts, figures, and reference list.

Reviewer #2 (Remarks to the Author):

The revised manuscript has fully considered the comments of the reviewers. Current version highlights the significance of the proposal and the methodology is well presented.

It is suggested to explain the meaning of ccc in Figure 1c.

Response: An explanation of ccc is added to the caption of Fig. 1.

It is suggested to mark the speckle pattern and its direction in Figure 2a.

Response: The speckle pattern shown in Fig. 2a remains unchanged. We assume the reviewer intended to add speckle patterns corresponding to different positions of the point source and indicate the shift direction in Fig. 2b. However, compared with displaying multiple speckle patterns, the current figure is sufficiently accurate and is more concise. Hence, we decide to keep the figure in the current status.

It is suggested to check whether N_bg is N_gt in the sentence 'N_bg is the total pixel count in Y_gt', below Figure 7.
Response: Thanks for pointing out the typo. We have corrected it in the manuscript update.

Reviewer #3 (Remarks to the Author):

The authors have addressed the major technical and conceptual concerns. The revised manuscript clarifies the physical interpretation of the learned inverse mapping. The discussion of ballistic and broadband components has been refined, and the experimental limitations are now more explicitly addressed.

Reviewer #3 (Remarks on code availability):

The code on GitHub has detailed README file with detailed instructions for installing and running.
(https://github.com/XuyuZhang98/Code-for-imaging-through-scattering-media-based-on-deep-learning.git)

The authors also provided a Code Ocean capsule; however, it appears to be set to private, preventing reviewer-accessible execution.

Response: Sorry for the technical issue. During our previous submission, there were no restrictions for open access. Nevertheless, we have reuploaded the code, and we hope the matter is now cleared.
